# Properties of encapsulated raspberry powder and its efficacy for improving the color stability and amino acid composition of pastırma cemen pastes with different pH during long term cold-storage

**DOI:** 10.1007/s13197-024-06029-6

**Published:** 2024-07-20

**Authors:** Muhammet Irfan Aksu, Nevzat Konar, Emre Turan, Faruk Tamtürk, Arda Serpen

**Affiliations:** 1 Faculty of Agriculture, Department of Food Engineering, Atatürk University, Erzurum, 25100 Türkiye; 2 Faculty of Agriculture, Department of Dairy Technology, Ankara University, Ankara, 06170 Türkiye; 3 Faculty of Agriculture, Department of Food Engineering, Ordu University, Ordu, 52200 Türkiye; 4 DÖHLER Food, İstanbul, Turkey

**Keywords:** Cemen paste, Encapsulated raspberry powder, Bioactive compounds, pH, Amino acid composition, Color

## Abstract

In this study, the changes in the physicochemical properties, color stability, and amino acid composition of cemen paste (CP) produced by adjusting to different pH levels (3.0, 4.0, 5.0, and 6.0) and enriched with different amounts (0%, 3%, 4% and 5%) of encapsulated raspberry powders (ERP) during 120 days of storage at 2.0 ± 0.5 °C were investigated. ERP had an important potential for use as a food ingredient with its bioactive components (total phenolic content (TPC), anthocyanin and ascorbic acid), antioxidant activity and high redness values. Essential and non-essential amino acid composition, moisture content, pH, lightness (L*), redness (a*), yellowness (b*), chroma (C*) and hue angle (^o^*h)* values of cemen pastes (CPs) were significantly (*p* < 0.01) affected by initial pH of CP (I-pH), ERP levels (ERPL) and storage time (ST) parameters and all possible interactions. The inclusion of ERP contributed to the improvement of CP visual properties and color stability by increasing redness. CPs with lower I-pH values had higher a* values and higher color stability over the storage period. In general, increasing ERPL and decreasing I-pH resulted in decreased amino acid contents of CPs. In conclusion, acidification (3.0, 4.0) of I-pH of CP and using ≥ 4.00% ERP can be recommended as an effective way to provide more stable samples based on color characteristics and amino acid content.

**Graphical abstract:**

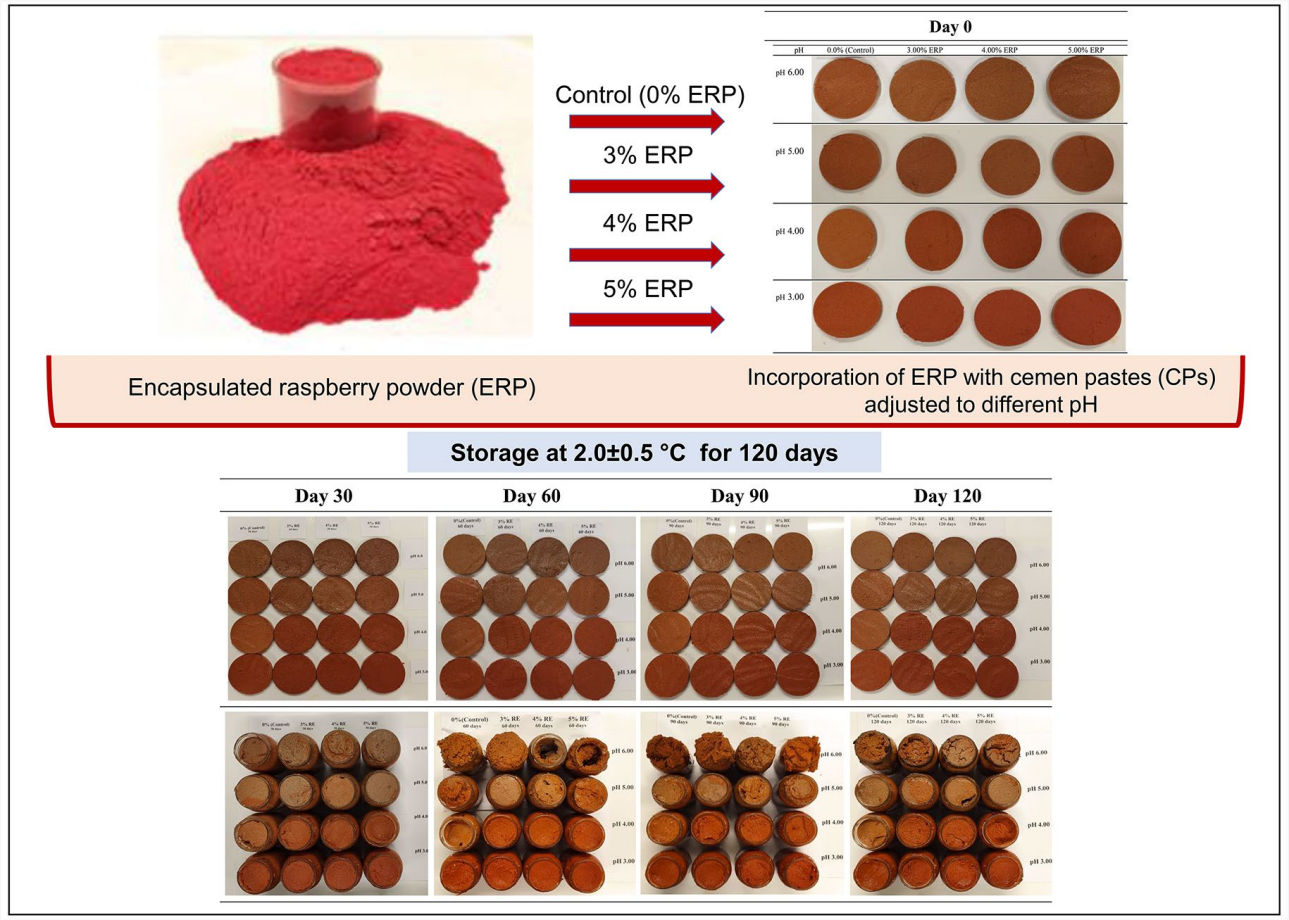

**Supplementary Information:**

The online version contains supplementary material available at 10.1007/s13197-024-06029-6.

## Introduction

“Cemening” is a crucial application to produce pastırma with unique taste, flavour and visual properties. However, the composition and various properties (such as pH) of cemen paste should be considered for the stability and quality of pastırma. Conventionally, *Trigonella foenum graecum* seed flour, hot red pepper powder, sweet red pepper powder, garlic and water are used to produce CP, and the color of CP is formed depending on the quality and characteristics of the red pepper (Aksu et al. 2016, 2017, 2020a). However, to satisfy consumer expects, various coloring agents may be used by producers, even synthetic agents. Using them has both a possible food safety risk and is against national and international regulations. For this reason, determination of natural coloring agents and investigation of their usage effects on final product is needed.

Considering this issue, some studies have been performed recently. These recent studies revealed that freeze-dried raspberry water extracts are potential natural coloring agents for CP to improve visual properties (Aksu et al. 2021, 2022). In other words, this novel ingredient containing polyphenols, especially anthocyanins, has great potential to improve both the technological (color and color stability, etc.) and functional (antioxidant activity, etc.) properties of CP and pastırma. Pre-treatments for these applications (extracts production) are carried out with different solvents such as water, methanol and petrolium ether (Aksu and Derman 2022). However, there are low-cost alternatives for freeze-drying. One of these alternatives is spray drying, which also has advantages for scale-up and industrial applications (Konar et al. 2022). For this reason, raspberry juice samples were encapsulated by using spray-dryer and maltodextrin as a widely used carrier agent.

Regarding food coloring, the various physico-chemical properties of related food matrices should be considered. These properties affect both the color and color intensity of the final product, as well as the visual stability throughout the process and shelf life. In addition, the coloring effects of pigments may depend on the ambient pH. For instance, the use of anthocyanins as coloring agents result from red to blue depending on pH (Jensen et al. 2011). Main pigments of raspberry are anthocyanins. Therefore, the coloring effect and color stability of raspberry anthocyanins in different-pH cemen pastes (CPs) pre- and post-pastırma application should be determined by considering final product quality. In previous studies, these interactions were not considered for both CP and pastırma.

CP composition affects many quality characteristics of pastırma as well as the free- and total amino acid content and profile (Ceylan and Aksu 2011; Erdemir and Aksu 2017). The high content of glutamic acid, arginine, lysine and leucine (El-Mahdy and El-Sebaiy 1985) of *Trigonella foenum graceum* L. seed flours used in the composition of CP causes an increase in the amount of these amino acids in pastırma as a result of cemening (Deniz et al. 2016; Erdemir and Aksu 2017; Aksu and Erdemir 2022). The pH value of CP affects many quality properties, stability and bioavailability of bioactive components, visual characteristics, even texture and sensory properties (Aksu et al. 2020a). In this context, various organic acids can be used to modify the pH of CP. Considering its use as an ingredient in meat products and its possible effects, lactic acid stands out as a good choice. In any case, modifying the pH of CP may potentially affect structural properties such as changes in proteins and formation of free amino acids. However, there are limited studies on this subject (Aksu et al. 2020a). Therefore, there is a need to investigate the interaction between pH and natural pigment sources such as encapsulated raspberry powders (ERP).

The aims of this study were to determine the effects of ERP usage and pH levels on the properties of pastırma CP during long-term cold storage, especially color stability and amino acid composition. To our knowledge, this is the first study to determine the effects of using various amounts of ERP as anthocyanins source on the color, amino acids and some other quality properties of CP with different pH values. In addition, the effects of storage time and the interaction between these variables were investigated in the present study. Moreover, amino acid composition, bioactive compounds and antioxidant capacity of ERP were determined for its possible use in the composition of various food products in terms of nutritional quality.

## Materials and methods

### Materials

*Trigonella foenum graecum* seed flour, hot red pepper powder, sweet red pepper powder, garlic were procured from Namsan Food Inc. (Eskişehir, Türkiye). Raspberry (*Rubus ideaus* L.) juices and maltodextrin (12–16 DE) were provided by Dohler Food R&D Center (Karaman, Türkiye).

### Encapsulation of raspberry juices

**Fig. 1:**
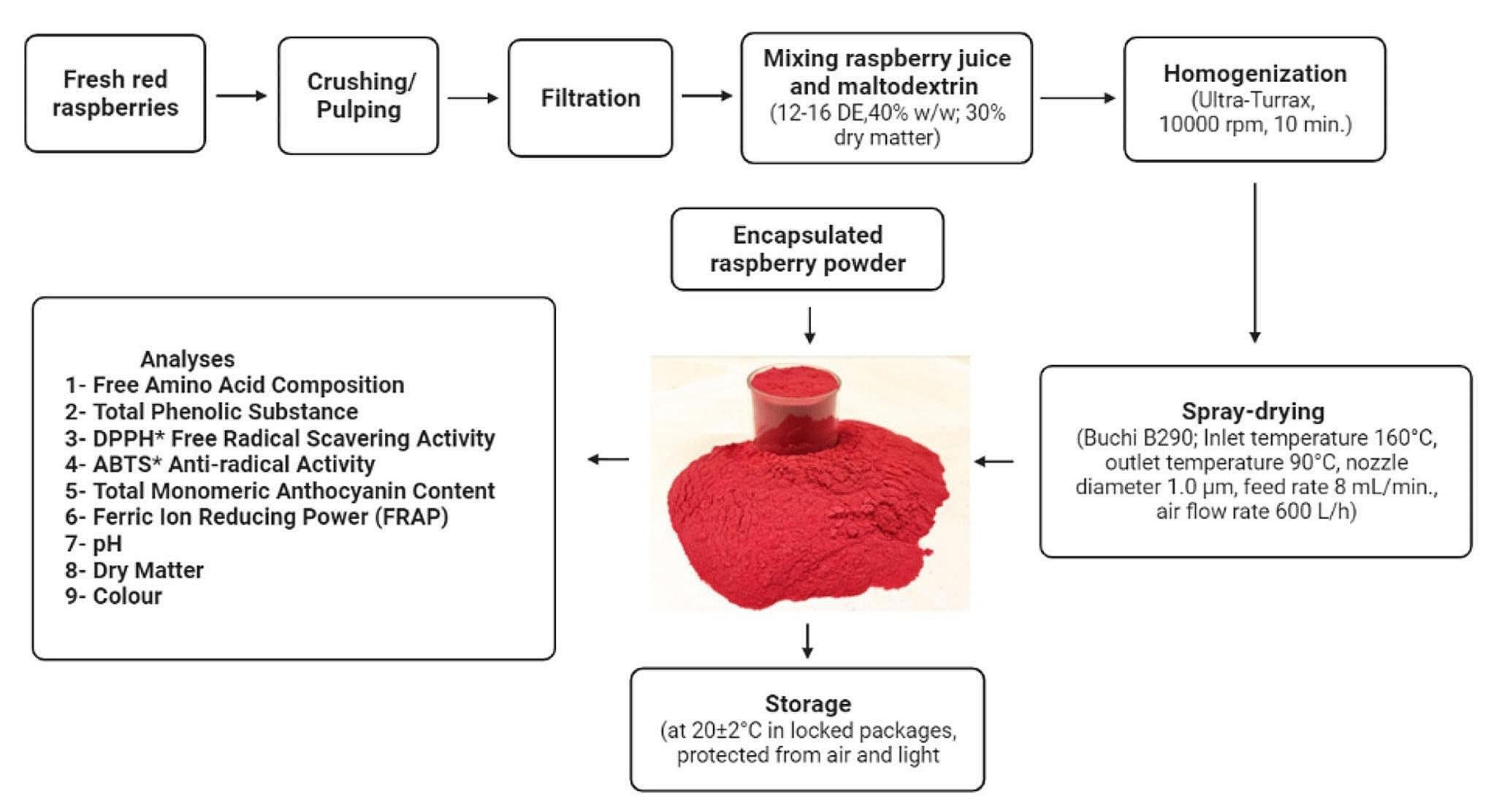
Sample preparation method for encapsulated raspberry powder with spray drying

Red raspberry (*Rubus ideaus* L.) juices were encapsulated as described in Fig. 1. Spray drying were carried out by using red raspberry juice (14.0°Bx) and maltodextrin (12–16 DE, Syral Company, Marckolsheim, France) in a lab-scale spray dryer (B290, Buchi, Fluwil, Switzerland). For process, maltodextrin was added to red raspberry at 25 °C (juice: maltodextrin, 1:1). Then, continuous mixing was carried out at 10,000 rpm for 10 min using Ultra-Turrax homogenizer (IKA T25 Ultra-Turrax, Staufen, Germany). Drying conditions were as 6 bar gas pressure, 1.40 mL min^− 1^ flow and 65 mbar atomization pressure. Spray dryer inlet and outlet temperature values were 160 °C and 90 °C, respectively. The dried samples were stored at 20.0 ± 2.0 °C in air tight dark color packages (Gagneten et al. 2019; Aksu et al. 2023).

### ERP characterization

Ethanol and water extracts of ERP were used to determine bioactive compound content and antioxidant activity. For the extraction of bioactive compounds, 0.5 g ERP was mixed with 5 mL acidified ethanol: distilled water (80:20 v/v) containing 0.1% HCl or distilled water. Subsequently, the mixtures were shaken on an MR-12 Rocker Shaker (Biosan, Latvia) at 50 rpm for 4 h at room temperature. After shaking, the mixtures were centrifuged at 2900 x *g* for 10 min and the supernatants were decanted. This process was repeated for the residue and the extracts obtained were used for total phenolic content (TPC), total anthocyanin content and antioxidant activity assays. The TPC of ERP samples in gallic acid equivalents (GAE) was determined according to the Folin-Ciocalteu procedure (Singleton et al. 1999). For total anthocyanin content, the pH-differential method described by Lee et al. (2005) was followed. The ascorbic acid (vitamin C) content of ERP samples was determined based on a spectrophotometric method.

Antioxidant activity of ERP was determined by using three different assays. DPPH antiradical activity (DPPH-ARA) was determined by applying the method given by Blois (1958). ABTS antiradical activity (ABTS-ARA) and ferric ion reducing antioxidant power (FRAP) were determined according to the methods given by Re et al. (1999) and Benzie and Strain (1996), respectively. All antioxidant activity results were expressed as trolox equivalent (TE).

The amino acid profile of ERP was determined using LC-MS/MS (Shimadzu, Kyoto Japan) (Henderson et al. 1999; Ceylan and Aksu 2011; Aksu and Erdemir 2022). The moisture content was determined on the basis of weight loss after drying in an oven. L* (lightness), a* (redness), b* (yellowness), C* (chroma) and ^o^*h* (hue angle) instrumental color values were recorded with a colorimeter device (CR-400, Minolta Co, Osaka, Japan). The pH was measured with a benchtop pH-meter (Hanna Instruments, Bedfordshire, UK) calibrated with buffer solutions.

### Production of cemen paste

Cemen paste (CP) samples were prepared according to methods used by Aksu and Kaya (2005); Aksu et al. (2020b). Sample compositions were as follows; 500 g cemen flour (*Trigonella foenum graecum* seed), 350 g fresh garlic, 75 g powdered paprika, 75 g powdered hot red pepper and 1100 mL water. CPs were initially divided into 4 groups and their pH values were adjusted to 5.00, 4.00 and 3.00 by using lactic acid (TK200640.01001, extra pure, food-grade, %80–85, Tekkim, Türkiye), whereas CP without this organic acid was the control sample (pH ~ 6.00) (Supplementary Material-1). Then, each of CPs were enriched by adding ERP at various amounts [0.00% (control for ERP), 3.00%, 4.00% and 5.00%, m/m]. Each CP sample was deposited sterilized jars (250 mL) and stored at 2.00 ± 0.5 °C for 120 days. During storage, the pH and colorimetric (L*, a*, b*, C* and º*h*) analyses were performed at 0., 30., 60., 90. and 120. days of storage. In addition, moisture content and amino acid composition were determined at the initial (0th Day) and end (120th Day) of storage.

### Cemen paste analyzes

#### Determination of pH values

The pH values of the samples were determined using a pH meter according to method used by Aksu et al. (2020a).

#### Determination of moisture content

The moisture content of samples (10 g) weighed in nickel cups was determined by drying at 100–102 °C in a drying cabinet until constant weight (16–18 h).

#### Color analysis

The color characteristics (L*, a*, b*, C* and ^o^*h*) of the ERP and CP samples were determined by using a colorimeter (CR-400, Minolta Co, Osaka, Japan) and CIE-Lab (Commision Internationalele de I’e Clairage) method (Aksu et al. 2020a).

#### Amino acid content and profile

The amino acids amounts and profile of CP and CP ingredients were determined according to the LC-MS/MS (Shimadzu, Kyoto Japan) method used by Henderson et al. (1999), Ceylan and Aksu (2011) and Aksu and Erdemir (2022) as mg/100 g in dry matter.

### Statistical analysis

The study was carried out according to the completely randomized block design with three replications. Analysis of variance was performed using the SPSS package program (SPSS 20.0). Averages of the main sources of variation were compared with the Duncan Multiple Comparison Test. In trial, as main effects of independent variables as CP I-pH levels (6.00, 5.00, 4.00 and 3.00), ERP usage amount (0.0, 3.0, 4.0 and 5.00%, m/m) and storage time (0, 30, 60, 90 and 120 days) were investigated.

## Results and discussion

### Characteristics of ERP

Some properties, bioactive compound content and antioxidant capacity of ERP are given in Table 1. In encapsulation and drying studies, various parameters can be considered for yield and efficiency determination. For efficiency, the amount of pigment, bioactive component, macro- or micro-other components are taken into account. The total mass change at the process input and output is used for yield determination. In this study, encapsulation yield taking into account losses during the process and determined based on the total dry matter (Konar et al. 2022), was determined to be 78.4% ± 2.55. This value was consistent with previous studies where biomass containing pigment was encapsulated using maltodextrin through the spray dryer method (Bustamante et al. 2016; Konar et al. 2022).

**Table 1:** Some properties, bioactive compound content and antioxidant capacity of encapsulated raspberry powders (ERP)

Parameter	ERP Ethanol Extract	ERP Water Extract	ERP
Total phenolic content (mg GAE/100 g)	663.1 ± 28.7^a^	579.8 ± 8.75^b^	-
Total anthocyanin content (mg cyanidine 3-glucoside/100 g)	158.6 ± 1.67^a^	57.9 ± 5.10^b^	-
DPPH* antiradical activity (µg TE/mg)	11.7 ± 1.41^a^	10.2 ± 1.04^a^	-
ABTS* antiradical activity (µg TE/mg)	16.3 ± 0.73^a^	15.1 ± 0.54^b^	-
Ferric reducing antioxidant power (FRAP) (µg TE/mg)	277.7 ± 16.2^a^	195.1 ± 17.2^b^	-
Ascorbic Acid (mg/kg)			337.1 ± 8.23
Moisture content (g/100 g)			4.48 ± 0.02
pH			3.29 ± 0.01
L*			45.7 ± 0.48
a*			46.3 ± 0.13
b*			14.0 ± 0.18
Chroma			48.4 ± 0.18
Hue Angle			16.8 ± 0.17
**Amino Acids (mg / 100 g**, **dry matter)**			
Alanine			555.5 ± 1.36
Arginine			126.8 ± 0.30
Aspartic Acid			383.0 ± 0.44
Cystine			147.7 ± 0.23
Glutamic Acid			574.2 ± 1.32
Glycine			504.4 ± 0.66
Histidine			76.6 ± 0.12
Isoleucine			78.6 ± 0.03
Leucine			81.2 ± 2.29
Lysine			206.0 ± 0.44
Methionine			0.00 ± 0.00
Ornithine			67.6 ± 0.06
Phenylalanine			179.0 ± 0.71
Proline			207.7 ± 0.84
Serine			407.2 ± 0.61
Threonine			10.3 ± 0.27
Tyrosine			43.7 ± 0.04
Valine			205.1 ± 0.23

Mean ± Standard Deviation

L*, a* and b* values of ERP were determined as 45.7 ± 0.48, 46.3 ± 0.13, and 14.0 ± 0.18, respectively. In a recent study, similar a* values (46.95 ± 2.05) were determined for raspberry extracts produced by freeze-drying method (Aksu et al. 2021), but L* (36.77) and b* (18.23) values were found to be lower than the present study findings. These may be result of using maltodextrin as encapsulating agent, as well as spray-drying as an encapsulation and drying process at relatively high temperature. Various pigments and their sources can be encapsulated by spray-drying to improve their usage possibilities as coloring agent in foods. In addition, high a* values indicated that ERP had high redness.

The solvent used in the extraction and its polarity are effective in the recovery of phenolic compounds (Boeing et al. 2014). Main pigments of raspberry can be accepted as anthocyanins. Anthocyanins are soluble in water and alcohols, but insoluble in apolar organic solvents. Therefore, commonly used solvents for anthocyanin extraction are water, ethanol, methanol, acetone, and their mixtures (Burdulis et al. 2008; Boeing et al. 2014). However, considering the toxicity, environmental pollution and costs, ethanol has been proposed as a more suitable solvent for the extraction of bioactive compounds compared to methanol (Teng et al. 2013; Celant et al. 2016). It is also known that the addition of hydrochloric, acetic, formic or tartaric acids to the extraction solvent at low ratios increases the extraction efficiency of anthocyanins (Burdulis et al. 2008).

In the present study, the ethanol extract of ERP had significantly higher (*p* < 0.05) TPC and anthocyanin content than the water extract (Table 1). Similarly, ethanol extract of ERP exhibited stronger (*p* < 0.05) antioxidant activities (except DPPH-ARA) compared to water extract. These results are consistent with studies reporting that ethanol as a solvent is more effective for the extraction of polyphenols and anthocyanins compared to distilled water (Celant et al. 2016; Su et al. 2023). Similar to our findings, previous studies reported a strong correlation between the antioxidant potential of berries and their TPC and anthocyanin content (Boeing et al. 2014; Sadowska et al. 2020; Lebedev et al. 2022).

The anthocyanin content (57.9-158.6 mg cyanidin 3-glucoside/100 g dm) and TPC (579.8-663.1 mg GAE/100 g dm) of water and ethanol extracts of ERP were similar to values reported for spray-dried raspberry powders in previous studies (Aksu and Arslan 2024; Aksu et al. 2023; Sadowska et al. 2020), but higher than the contents determined by Gagneten et al. (2019) and Syamaladevi et al. (2012). The amount of ascorbic acid in ERP was 337.10 ± 8.23 mg/kg. Regarding antioxidant activity, DPPH-ARA, ABTS-ARA and FRAP values of ethanol and water extracts of ERP were determined in the range of 10.2–11.7, 15.1–16.3 and 195.1-277.7 as µg TE per mg sample, respectively. The antioxidant potential of ERP can be attributed to its rich content of bioactive compounds, including phenolic compounds (anthocyanins, phenolic acids, etc.) and ascorbic acid (Lebedev et al. 2022; Marino et al. 2024). As stated by Marino et al. (2024), freeze-dried raspberry powder (moisture: %6.1 ± 0.3) is an important source of nutrients and bioactive compounds. The researchers also reported that freeze-dried raspberry powder is a good source of polyphenol compounds such as proanthocyanidins, ellagitannins (sanguiin H-6, lambertianin C, and sanguiin H-10 isomers), anthocyanins (cyanidin-3-sophoroside: 406.3 ± 15.0 mg/100 g, cyanidin-3-glucoside: 93.7 ± 3.3 mg/100 g, and cyanidin-3-sambubioside: 18.1 ± 0.6 mg/100 g), and phenolic acids.

On the other hand, the TPC, anthocyanin and ascorbic acid contents of ERP were lower than those reported in the literature for freeze-dried raspberry powders (Marino et al. 2024; Aksu et al. 2021; Sadowska et al. 2020). This can be attributed to the lower juice content in ERP due to the presence of encapsulation agents (40%). In addition, ascorbic acid can degrade due to temperature exposure and processing time (Badin et al. 2020). When discussing these results, the difference in encapsulation and drying methods should be considered. There are also significant differences in the composition of the powdered products depending on the methods used to obtain powder from fruits and vegetables (Syamaladevi et al. 2012), geographical and botanical origin (Lebedev et al. 2022) and pre- and post-harvest conditions (Evdokimenko et al. 2021). In a previous study, high-pressure homogenization resulted decrease in a* values of raspberry powder encapsulated with gum arabic, whereas total anthocyanin, phenolic contents and total antioxidant activity were increased (Syamaladevi et al. 2012). Extracts produced by the freeze-drying method, which is advantageous in preserving the structure and bioactive compounds of foods to a large extent, are prone to stickiness and color loss due to their sugar content and glassy properties. Additionally, this method takes a long time and is costly (Sadowska et al. 2020). These problems do not occur in powders produced by the spray-drying method, and the physical structure and stability of bioactive compounds are preserved during storage (Gagneten et al. 2019; Konar et al. 2022).

Red raspberry is an acidic fruit and therefore the pH values of raspberry powders are also quite low. In the present study, the pH value of ERP (3.29) was determined similar to water extracts of freeze-dried and raspberry powder samples (3.36, 3.32) (Aksu and Arslan 2024; Aksu et al. 2021). The low pH value in extracts and powders can be accepted as an advantage by considering possible applications in food technology, such as CP production and coloring capacity and stability of anthocyanins. The free amino acids composition of the ERP is given also in Table 1. It was determined that the major ERP amino acids were glutamic acid > alanine > glycine > serine > aspartic acid > proline > lysine > valine.

### Cemen Paste enhanced with ERP

#### Physico-Chemical properties

The pH value for conventional CP is approximately 6.00 (Aksu et al. 2006). By considering usage in pastirma production, this environmental condition is a disadvantage for anthocyanins which coloring affects depends pH value. Therefore, CP samples at various pH values were prepared to investigate usage possibility and effects of ERP as an anthocyanin source. As it is known, anthocyanins are a special natural pigments that may provide bright red color depends on environmental conditions. Due to these properties, it is stated that anthocyanins can be used as an alternative to synthetic dyes in coloring many foods (Aksu et al. 2020a). The initial CP pH levels (I-pH), ERP usage and amounts (ERPL), the storage time (ST), I-pH x ERPL, I-pH x ST, ERPL x ST and I-pH x ERPL x ST interactions had significant effects on the final pH values of CP (*p* < 0.01) (Table 2). The effects of enhancement with ERP on pH values varied according to the initial pH. For samples with initial pH 5.0 and 6.0, pH values decreased by using ERP (*p* < 0.05) at the beginning of storage (Day 0), whereas there were not significant effects on other samples’ pH values. This result might be related to the increased buffering capacity of CPs, thanks to the lactic acid added to lower the pH. In this case, since the buffering capacity of CP increases, ERP usage cannot result with decreased pH, even they have low pH and high titration acidity. A similar resulted has been determined for CP including various amounts (0.0%, 0.8%, 1.0% and 1.2%) of freeze-dried red cabbage water extracts with different pH (6.0, 5.5, 5.0, 4.5 and 4.0) (Aksu et al. 2020a). In this previous study, the pH of CP with initial pH as 4.0 increased by about 0.1 unit with the addition of extracts, while the pH values of CP with initial pH 5.0, 5.5 and 6.0 decreased.

**Table 2:** pH, color properties and moisture content of cemen paste samples including various amounts of encapsulated raspberry powders (ERP)

	L*	a*	b*	Chroma (C*)	Hue angle (^o^h)	pH	Moisture Content (g/100 g)
***Initial pH***	***Initial pH of cemen pastes (I-pH)***						
3.00	43.4 ± 2.26^b^	25.4 ± 1.26^a^	37.5 ± 2.18^a^	45.3 ± 2.05^a^	55.9 ± 1.80^d^	3.09 ± 0.08^d^	60.26 ± 1.00^d^
4.00	44.4 ± 3.29^a^	22.4 ± 2.42^b^	37.0 ± 2.35^b^	43.3 ± 2.74^b^	59.0 ± 2.55^c^	4.01 ± 0.05^c^	61.16 ± 1.26^b^
5.00	44.0 ± 2.85^a^	17.6 ± 1.13^c^	32.6 ± 2.30^c^	37.0 ± 2.56^c^	61.6 ± 1.02^b^	4.91 ± 0.09^b^	62.91 ± 0.67^a^
6.00	43.4 ± 2.32^b^	15.0 ± 1.76^d^	29.6 ± 1.96^d^	33.2 ± 2.39^d^	63.2 ± 2.18^a^	5.26 ± 0.42^a^	60.50 ± 2.38^c^
SEM	0.153	0.052	0.116	0.120	0.079	0.003	0.049
P	**	**	**	**	**	**	**
***ERP***	***Encapsulated Raspberry Powder Levels (ERPL)***						
Control	46.4 ± 2.70^a^	18.6 ± 4.05^b^	34.9 ± 4.42^a^	39.5 ± 5.68^b^	62.2 ± 2.75^a^	4.33 ± 0.91^a^	62.12 ± 1.69^a^
3%	43.5 ± 2.00^b^	20.5 ± 4.35^a^	34.3 ± 3.82^b^	40.0 ± 5.45^a^	59.4 ± 2.61^b^	4.33 ± 0.88^a^	61.46 ± 1.48^b^
4%	43.1 ± 2.22^b^	20.5 ± 4.56^a^	34.4 ± 3.79^b^	40.1 ± 5.39^a^	59.4 ± 3.49^b^	4.31 ± 0.87^b^	60.78 ± 2.09^c^
5%	42.2 ± 2.06^c^	20.7 ± 4.47^a^	33.3 ± 3.59^c^	39.2 ± 5.30^b^	58.5 ± 3.21^c^	4.29 ± 0.84^c^	60.48 ± 1.45^d^
SEM	0.153	0.052	0.116	0.120	0.079	0.003	0.049
P	**	**	**	**	**	**	**
***Day***	***Storage Time (days***, ***ST)***						
0	46.3 ± 2.15^a^	22.0 ± 4.06^a^	36.8 ± 3.99^a^	42.9 ± 5.18^a^	59.1 ± 2.89^b^	4.43 ± 0.98^a^	61.44 ± 1.55^a^
30	44.7 ± 2.62^b^	20.3 ± 4.28^b^	35.1 ± 3.78^b^	40.6 ± 5.19^b^	60.2 ± 3.17^a^	4.36 ± 1.01^b^	nd
60	43.4 ± 2.24^c^	19.6 ± 4.45^c^	33.6 ± 3.37^c^	39.0 ± 5.02^c^	60.1 ± 3.60^a^	4.30 ± 0.87^c^	nd
90	42.4 ± 2.06^d^	19.3 ± 4.50^d^	32.9 ± 3.95^d^	38.2 ± 5.60^d^	60.1 ± 3.26^a^	4.27 ± 0.76^d^	nd
120	42.3 ± 2.36^d^	19.1 ± 4.35^d^	32.6 ± 2.97^d^	37.8 ± 4.68^e^	60.0 ± 3.97^a^	4.22 ± 0.71^e^	60.98 ± 1.99^b^
SEM	0.172	0.058	0.129	0.134	0.088	0.004	0.035
*P*	**	**	**	**	**	**	**
	***Interactions***						
*I-pH x ERPL*	**	**	**	**	**	**	**
*I-pH x ST*	**	**	**	**	**	**	**
*ERPL x ST*	**	**	**	**	**	**	**
*I-pH x ERPL x ST*	**	**	**	**	**	**	**

Mean ± Standard Deviation. Different lowercase letters (a-e) in the same column for each parameter indicate significant differences between the means (*P* < 0.05). nd: Not determined

As seen in Table 2, the average pH values were highest in the 5% ERP-added group, but similar in the control and 3% ERPL samples. The acidic pH (3.29) of ERP increased (*p* < 0.05) the pH values of the CP samples in the group with pH 3.0 compared to the control, while the pH values of the other pH groups (pH 4.0, 5.0 and 6.0) generally decreased (*p* < 0.05) (Supplementary Material 2). These changes were dependent on the increasing ERP concentration (*p* < 0.05). Moreover, the decrease in the pH values of CPs with ERP inclusion was more pronounced (*p* < 0.05) in the pH 5.0 and pH 6.0 groups.

On the other hand, significant (*p* < 0.01) effects of storage time on pH values were determined. pH values of CPs with initial pH 5.0, 4.0 and 3.0 increased during storage. However, contrary to these results, there was significant decrease for both control and ERP-containing CP samples with an initial pH of 6.0. These decreases were more pronounced after 60 days of storage, especially in control and 3.00% (m/m) ERP including CP (Supplementary Material 2). These can be observed clearly from the images of CP samples in Supplementary Material 1. The CP with initial pH 6.0 deteriorated faster after this storage period. Gas entrapment occurred in the jars in which the samples were kept, and CPs overflowed from the jar when the jars were opened for analysis due to gas entrapment. The decrease in pH of control CP with initial pH 6.0 at 120th day of storage was higher than ERP including samples (Supplementary Material 2). According to these results, ERP enhancement caused preservative effects for CP even with higher initial pH. This protective effect can be attributed to the antioxidant and antimicrobial properties of raspberry powder resulting from its rich phytochemical content. The ERP amounts should also be taken into account for this effect. The encapsulated extracts are more effective than non-encapsulated ones as a natural preservative in foods (Feridoni and Shurmasti 2020). Encapsulation with maltodextrin of ERP further increases this effect (Makhathini et al. 2022).

I-pH values (*P* < 0.01), ERPL (*p* < 0.01) and ST (*p* < 0.01) had significant effects on the moisture content of the CP samples. Higher moisture content was determined for CP with initial pH 5.0 (*p* < 0.05), and the moisture contents of control samples were higher than for the other sample groups (*p* < 0.05). Fruit and vegetable powders are strongly hydroscopic, and this poses a problem in industrial use. For this reason, the problem is solved by using anticaking agents such as maltodextrin, calcium silicate, tricalcium phosphate, and etc. in the production of powder from fruits and vegetables, and the water retention capacity of the produced powders decreases (Lipasek et al. 2012; Asgar et al. 2016; Wei et al. 2022). However, although maltodextrin was used in ERP production, by increasing the ERP amount, CP moisture contents decreased. These decreases may be due to both the low moisture content (Table 1) and water-absorption capacity of ERP, and the proportional increase of ERP in the CP composition.

#### Color properties

The changes in the color of CP produced within the scope of the research during the storage period are given visually in Supplementary Material (1) All main factors (I-pH value, ERPL, ST) and all possible two-way and three-way interactions of these parameters had a significant (*p* < 0.01) effect on all instrumental color parameters (L*, a*, b*, C* and ^*o*^*h*) of CP samples (Table 2). The changes in color values of CPs adjusted to different pH values and enriched with ERP during 120 days of storage are presented in Supplementary Material (2) L* values of CP with initial pH 4.00 and 5.00 were higher than samples with initial pH 3.0 and 6.0. L* values decreased depending on used ERP amount. These findings related with water binding effects of ERP in the CP composition. L* values decreased during storage for studied pH values and ERP amounts (*p <* 0.05). Especially for samples with initial pH 3.0 and including 4.00% and 5.00% ERP, the change in L* value during storage was lower than other samples (Supplementary Material 2). In previous studies, it was determined that CP L* values decreased as a result of decreasing pH (Aksu et al. 2020b) and increasing plant extract amount (Aksu et al. 2020a, b). The b* value, whose positive values indicate yellowness, was found to be lower in the samples with ERP added than the control, except for the samples with pH 4.0 at the beginning of storage (Day 0), and the b* value decreased due to the decrease in the pH of CP during storage (*p <* 0.05) (Supplementary Material 2).

As an important quality criterion for CP, higher a* values is related to the pH value and its effect on the pigments in the composition. The enrichment of CPs with ERP and the reduction of I-pH increased the redness (a*) values (Table 2). Regarding the change in the redness values of CPs during storage (Supplementary Material 2), the hierarchy for a* values and color stability during storage was pH 3.0 > 4.0  > 5.0 > 6.0. Increasing the ERP concentration in the pH 3.0 and 4.0 groups resulted in higher (*p* < 0.05) redness and colour stability during storage compared to the control samples. However, no positive effect of ERP inclusion on initial a* values was observed in CP groups with high pH (pH 5.0 and 6.0), and even lower (*p <* 0.05) values were obtained in ERP-added samples (Supplementary Material 2) At these pH values, the effect of ERP on the a* values was evident in the following days of storage, and the values determined in the samples with ERP added at the 30th, 90th and 120th days of storage were found to be higher than the control samples (*p <* 0.05). In other words, the inclusion of ERP contributed significantly to the maintenance of the colour of CPs with pH 6.0 and 5.0 during long-term cold storage (Supplementary Material 2). Aksu et al. (2020a) determined that the pH value was lower than 5.0 to reveal the effectiveness of coloring agents in pastırma CP, the effect of red pepper powder which main pigments source for conventional CP. They reported that the effect of red pepper on a* value in CP was higher at pH 4.0 and 4.5 than at pH 5.0, 5.5 and 6.0. They recommended that the effects of extracts on the color quality of CP with different pH should be investigated. The results of the present study corrected this suggestion by considering a* values of the samples with pH 6.0 and 3.0.

Previous studies have shown that natural colorants such as anthocyanins are more effective at CPs with low pH values (Aksu et al. 2020a, b, 2021). This situation is related to the change in the molecular structure of anthocyanins. Anthocyanins are more stable in acidic solutions (pH < 2) where the red flavylium cation (red color) predominates. Due to rapid deprotonation when pH rises from 2.0 to 4.0, purple or blue quinonoidal species are observed. The pH of pastırma CP is around 6.0, and between pH 4 and 6, the flavylium cation hydrates and is in the form of carbinol pseudobase (colorless) and chalcone (colorless/pale yellow). Also, at these pH values, quinonoidal base, carbinol pseudobase, and chalcone forms may coexist and a violet color may occur (Tang et al. 2019; Liu et al. 2021). A rising pH value makes anthocyanins more susceptible to attack by water and other nucleophilic molecules and leads to their degradation (Gamage and Choo 2023). On the other hand, the presence of copigment in the medium prevents the formation of a colorless falvylium cation and contributes to the maintenance of the color of anthocyanins (Babaloo and Jamei 2018). Other factors that affect the stability of the chemical structures of anthocyanins are oxidative degradation, solvents, sugars, vitamin C, temperature, light and the presence of metal ions (Babaloo and Jamei 2018; Liu et al. 2021). In the research conducted by Jin and Lee (2024), it was determined that the emulsion in which Gerdenia red was used as a natural colorant both increased the a* value and decreased the pH value, and these effects could be increased with the addition of maltodextrin and sodium caseinate.

According to these results, it is possible to state that the redness, which is important for pastırma CP, can be improved by decreasing the pH alone or can be increased by using ERP and also the storage stability can be improved. ERP containing polyphenols, especially anthocyanins, has great potential to improve color and color stability properties of CP. The better maintenance of colour during storage in CPs adjusted to lower pH groups can be attributed to the antioxidant potential of ERP and the greater stability of anthocyanins under acidic conditions. In a similar study investigating the stability of anthocyanin extracts adjusted to different pH values (pH 3.0–6.0) during 30 days cold storage (at 4 °C), the anthocyanin retention rate of black goji berry extracts at the end of storage was 70%, 39%, 33%, and 4% for pH 3.0, 4.0, 5.0 and 6.0, respectively, while these rates were 100%, 97%, 89% and 77% for extracts obtained from purple sweet potato, respectively (Gamage and Choo 2023). The researchers also reported that the anthocyanin extracts obtained from black goji berry and purple sweet potato showed higher stability in terms of phenolic content and antioxidant activity at pH 3.0 and pH 4.0.

#### Amino acid profiles

##### Cemen paste ingredients

Amino acid contents of CP main ingredients such as *Trigonella foenum graecum* seed flour, hot red pepper powder, and sweet red pepper powder are given in Table 3. It has been determined that there are significant differences in the amount of amino acids among the ingredient used in CP production. Except for aspartic acid, cysteine, glycine and ornithine, other amino acids were determined as higher than other ingredients for commercial *Trigonella foenum graecum* seed flour. As major aspartic acid source for CP was the hot red pepper powder, whereas ornithine, glycine and cysteine content of sweet red pepper powder remarked (Table 3).

**Table 3:** Amino acid composition (mg / 100 g, dry matter) of main ingredients of cemen paste

Amino acid	Hot red pepper powder	Sweet red pepper powder	Trigonella foenum graecum seed flour
Alanine	777.4 ± 0.35^c^	934.7 ± 0.45^b^	1187.0 ± 1.05^a^
Arginine	790.8 ± 0.28^c^	800.9 ± 0.32^b^	1549.9 ± 0.24^a^
Aspartic Acid	3360.0 ± 8.55^a^	2319 ± 0.54^c^	2718.4 ± 3.54^b^
Cystine	259.2 ± 1.32^c^	297.2 ± 0.16^a^	267.9 ± 1.22^b^
Glutamic Acid	2096 ± 1.53^c^	2234 ± 3.34^b^	4540.0 ± 10.67^a^
Glycine	1104 ± 6.44^c^	1441 ± 2.48^a^	1233.0 ± 0.62^b^
Histidine	331.0 ± 0.08^c^	384.9 ± 0.83^b^	700.1 ± 0.33^a^
Isoleucine	284.8 ± 0.73^c^	313.7 ± 0.18^b^	427.0 ± 0.52^a^
Leucine	679.8 ± 16.0^c^	826.3 ± 0.24^b^	1452.2 ± 13.3^a^
Lysine	767.0 ± 0.35^c^	833.2 ± 5.11^b^	1582 ± 2.30^a^
Methionine	57.8 ± 0.01^c^	115.5 ± 0.13^b^	167.8 ± 0.42^a^
Ornithine	104.5 ± 0.05^b^	114.4 ± 0.01^a^	104.0 ± 0.01^c^
Phenylalanine	543.2 ± 4.62^c^	613.6 ± 5.43^b^	915.6 ± 6.52^a^
Proline	736.4 ± 0.23^c^	873.3 ± 0.13^b^	1183 ± 0.19^a^
Serine	846.5 ± 0.49^c^	860.1 ± 0.01^b^	1421.0 ± 2.00^a^
Threonine	471.6 ± 0.21^c^	559.8 ± 0.31^b^	619.9 ± 1.54^a^
Tyrosine	258.8 ± 0.23^c^	277.6 ± 0.09^b^	396.8 ± 0.91^a^
Valine	516.5 ± 0.54^c^	533.4 ± 0.30^b^	575.1 ± 0.46^a^

Mean ± Standard deviation. Different lowercase letters (a-c) in the same row indicate significant differences between the means (*P* < 0.05)

##### Enhanced cemen pastes

Essential and non-essential amino acid contents of CP samples are given in Tables 4 and 5, respectively. The essential amino acid contents of CPs were significantly (*p* < 0.01) affected by I-pH, ERPL, ST and all the two- and three-way interactions of these factors, but ST had insignificant (*p* > 0.05) effect on the amount of phenylalanine (Table 4). Depending on the pH values of CPs, the amount of essential amino acids varied (*p <* 0.05) and the lower lysine, isoleucine, leucine, phenylalanine and valine contents were determined in samples with lower pH (pH 3.0). Whereas, the methionine and threonine contents of CP with pH 4.0 were higher than other samples. Generally lower essential amino acid contents were determined for low pH (3.00) samples than high pH (6.00). In a previous study, it was reported that nitrogen solubility changes depending on pH in protein concentrates obtained from *Trigonella foenum graecum* seed flour (El Nasri and El Tinay 2007). Researchers have determined that the minimum protein solubility was at pH 4.5 and solubility had tend to increase at acidic and basic values. Also, in the current study, solubility characteristics might cause the low of amount of amino acids in CP with low pH.

**Table 4:** Essential amino acid composition of cemen paste samples including ERP (mg/100 g, dry matter)

Variable	Lysine	Isoleucine	Leucine	Methionine	Phenylalanine	Valine	Threonine
***Initial pH of cemen pastes (I-pH)***							
**3.00**	1217 ± 301^c^	404.0 ± 124^d^	1087 ± 144^d^	128.0 ± 22^d^	895.7 ± 91^d^	802.9 ± 105^d^	704.4 ± 254^c^
**4.00**	1429 ± 230^b^	502.8 ± 87^b^	1277 ± 285^b^	158.5 ± 41^a^	1036 ± 152^b^	957.9 ± 141^c^	826.5 ± 355^a^
**5.00**	1482 ± 204^a^	495.8 ± 158^c^	1223 ± 272^c^	136.0 ± 49^c^	1003 ± 125^c^	984.8 ± 197^b^	791.5 ± 337^b^
**6.00**	1479 ± 122^a^	555.5 ± 70^a^	1299 ± 173^a^	150.7 ± 65^b^	1057 ± 96^a^	1030 ± 123^a^	791.5 ± 302^b^
***SEM***	1.935	1.105	5.538	0.178	3.976	1.106	0.981
***P***	**	**	**	**	**	**	**
***Encapsulated Raspberry Powder Levels (ERPL)***							
**0.00%**	1466 ± 332^a^	491.9 ± 168^b^	1306 ± 338^a^	139.2 ± 54^d^	1032 ± 191^a^	959.3 ± 239^a^	820.2 ± 392^a^
**3.00%**	1422 ± 257^b^	472.5 ± 163^d^	1192 ± 253^bc^	140.8 ± 43^c^	1007 ± 153^b^	923.6 ± 157^c^	783.4 ± 248^b^
**4.00%**	1336 ± 90^d^	485.0 ± 38^c^	1181 ± 128^c^	143.1 ± 44^b^	958.7 ± 64^d^	934.2 ± 122^b^	737.1 ± 329^d^
**5.00%**	1383 ± 235^c^	508.7 ± 93^a^	1205 ± 171^b^	150.2 ± 51^a^	994.8 ± 75^c^	958.5 ± 135^a^	773.2 ± 278^c^
***SEM***	1.935	1.105	5.538	0.178	3.976	1.106	0.981
***P***	**	**	**	**	**	**	**
***Storage Time (days) (ST)***							
**0**	1512 ± 120^a^	506 ± 47^a^	1181 ± 126^b^	110.4 ± 22^b^	1001 ± 84^a^	897.8 ± 63^b^	503.5 ± 73^b^
**120**	1292 ± 285^b^	473 ± 170^b^	1262 ± 307^a^	176.2 ± 43^a^	994.4 ± 167^a^	990.0 ± 218^a^	1054 ± 185^a^
***SEM***	1.368	0.781	3.916	0.126	2.811	0.782	0.694
***P***	**	**	**	**	NS	**	**
***Interactions***							
*I-pH x ERPL*	**	**	**	**	**	**	**
*I-pH x ST*	**	**	**	**	**	**	**
*ERPL x ST*	**	**	**	**	**	**	**
*I-pH x ERPL x ST*	**	**	**	**	**	**	**

ERP: Encapsulated raspberry powders. Mean ± Standard deviation. Different lowercase letters (a-d) in the same column for each parameter indicate significant differences between the means (*P* < 0.05). **: *P* < 0.01, NS: *P* > 0.05

**Table 5:** Non-essential amino acid composition of cemen paste samples including encapsulated raspberry powders (ERP) (mg / 100 g, dry matter)

Variable	Alanine	Arginine	Aspartic Acid	Cystine	Glutamic Acid	Glycine	Histidine	Ornithine	Proline	Serine	Tyrosine	
***Initial pH of cemen pastes (I-pH)***												
**3.00**	939.9 ± 400^d^	1571 ± 203^c^	2319 ± 254^d^	419.9 ± 60^d^	3486 ± 421^d^	1368 ± 474^d^	577.6 ± 59^d^	314.5 ± 104^c^	1127 ± 82^c^	1302 ± 141^d^	476.2 ± 58^d^	
**4.00**	1041 ± 420^c^	1847 ± 343^b^	2637 ± 477^a^	451.0 ± 43^b^	4075 ± 522^a^	1551 ± 406^b^	649.7 ± 151^a^	317.8 ± 85^b^	1274 ± 157^b^	1421 ± 163^b^	513.4 ± 86^b^	
**5.00**	1091 ± 365^b^	1847 ± 190^b^	2335 ± 347^c^	457.3 ± 53^a^	3940 ± 524^c^	1526 ± 458^c^	593.1 ± 112^c^	303.9 ± 67^d^	1271 ± 117^b^	1409 ± 164^c^	500.2 ± 87^c^	
**6.00**	1120 ± 392^a^	1858 ± 179^a^	2464 ± 272^b^	424.9 ± 69^c^	3968 ± 380^b^	1566 ± 458^a^	617.7 ± 55^b^	322.8 ± 70^a^	1286 ± 106^a^	1456 ± 198^a^	550.2 ± 61^a^	
***SEM***	1.647	2.253	3.385	1.309	5.013	3.375	1.804	0.415	1.448	1.817	0.696	
***P***	**	**	**	**	**	**	**	**	**	**	**	
	***Encapsulated Raspberry Powder Levels (ERPL)***											
**0.00%**	1072 ± 392^a^	1878 ± 407^a^	2533 ± 499^a^	428.3 ± 57^b^	4048 ± 775^a^	1624 ± 510^a^	661.1 ± 177^a^	303.1 ± 65^d^	1283 ± 203^a^	1464 ± 189^a^	542.1 ± 94^a^	
**3.00%**	1060 ± 422^b^	1812 ± 241^b^	2443 ± 371^b^	463.2 ± 57^a^	3933 ± 516^b^	1526 ± 420^b^	591.6 ± 74^c^	313.5 ± 80^c^	1249 ± 113^b^	1402 ± 217^b^	530.2 ± 97^b^	
**4.00%**	1036 ± 365^c^	1692 ± 113^d^	2355 ± 306^d^	429.8 ± 65^b^	3689 ± 188^d^	1426.7 ± 354^c^	585.4 ± 59^d^	316.6 ± 93^b^	1203 ± 94^d^	1365 ± 111^c^	480.4 ± 52^d^	
**5.00%**	1025 ± 421^d^	1741 ± 178^c^	2424 ± 244^c^	431.9 ± 50^b^	3800 ± 324^c^	1435 ± 497^c^	599.9 ± 29^b^	325.9 ± 90^a^	1223 ± 90^c^	1356 ± 155^d^	487.2 ± 33^c^	
***SEM***	1.647	2.253	3.385	1.309	5.013	3.375	1.804	0.415	1.448	1.817	0.696	
***P***	**	**	**	**	**	**	**	**	**	**	**	
	***Storage Time (days) (ST)***											
**0**	1419 ± 83^a^	1802 ± 136^a^	2536 ± 236^a^	485.5 ± 26.7^a^	3913 ± 305^a^	1891.8 ± 155^a^	579.8 ± 44^b^	240.9 ± 25^b^	1195 ± 70^b^	1505 ± 101^a^	510.2 ± 64^a^	
**120**	677.6 ± 143^b^	1759 ± 348^b^	2342 ± 441^b^	391.1 ± 39.1^b^	3822 ± 654^b^	1114 ± 262^b^	639.2 ± 134^a^	388.6 ± 38^a^	1285 ± 164^a^	1289 ± 164^b^	509.8 ± 89^a^	
***SEM***	1.165	1.593	2.394	0.926	3.545	2386	1.275	0.293	1.024	1.285	0.492	
***P***	**	**	**	**	**	**	**	**	**	**	NS	
	***Interactions***											
*I-pH x ERPL*	**	**	**	**	**	**	**	**	**	**	**	
*I-pH x ST*	**	**	**	**	**	**	**	**	**	**	**	
*ERPL x ST*	**	**	**	**	**	**	**	**	**	**	**	
*I-pH x ERPL x ST*	**	**	**	**	**	**	**	**	**	**	**	

ERP: Encapsulated raspberry powders. Mean ± Standard deviation. Different lowercase letters (a-d) in the same column for each parameter indicate significant differences between the means (*P* < 0.05). **: *P* < 0.01, NS: *P* > 0.05

As a result of ERP enhancement, lysine, leucine, phenylalanine, valine and threonine contents significantly decreased (*p <* 0.05). However, isoleucine and methionine were higher than the samples including 5.00% (m/m) ERP (*p <* 0.05). After 120 days storage, it was determined that only lysine and isoleucine amounts decreased (*p <* 0.05), while there was an increase for the other amino acids. According to these results, storage time had significant effects on the free essential amino acid contents, excluding phenylalanine (Table 4).

Lysine content of samples had relation pH and ERP amount. The higher loss was observed in the control and low ERP added samples at pH 3.0. This value is more stable for samples (pH > 4.0) including 4.00% and 5.00% (m/m) ERP. A similar trend was also determined for isoleucine. ERP had a protective effect on the structural degradation of lysine. Among the ingredients used in the CP content, the materials containing higher amount of lysine and isoleucine were commercial *Trigonella foenum graecum* seed flour, hot red pepper powder and sweet red pepper powder, respectively (Table 3). Lysine and isoleucine contents of ERP were 206.04 and 78.56 mg/100 g (in dm) (Table 1). For this reason, there was an increase in the amount of these amino acids of ERP-enhanced CPs. The increase in the leucine content during storage was determined for ERP-free CP (pH 4.0, 5.0 and 6.0). There was a higher increase in these CP samples including ERP. However, a decrease was determined for the control samples with pH 3.0 (Table 4). This might be a result of exposure to low pH environmental conditions. In addition, significant changes were determined for methionine as a result of storage (Table 4). Considering the results at the beginning of storage (Day 0), methionine content of CP samples with initial pH of 3.00, 4.00 and 5.00 increased depending on used ERP amount. Although methionine was not detected in ERP (Table 1), this effect in CP was due to the breakdown of cystine and thus the amino acid cysteine. Because methionine is formed as a result of the breakdown of cysteine. The decrease in cystine content during storage in all CP samples also confirmed this possible effect. The decrease of methionine content for CP with initial pH 6.00 (Table 4) indicates that cystine does not decompose at this pH and only methionine originating from the CP composition. During storage, a trend for valine content was determined as belongs to lysine, isoleucine, leucine and phenylalanine contents. The valine content decreased in control CP with initial pH 3.00, whereas increased valine contents were determined for other control samples (> pH 3.00).

In general, the amounts of valine determined at the end of storage in CP samples with initial pH 5.00 and 6.00 were higher than low pH samples (3.00 and 4.00). These might be related with the spoilage of the samples with these pH value at the end of storage (Supplementary Material 2) which indicates degradation in proteins, and increase amounts of various amino acids (Supplementary Material 3). Threonine contents increased remarkedly during storage. In this process, it is understood from the data obtained in the research that the pH of CP is effective in the increase in the amount of threonine, and the increase in the amount is higher as the pH increases. In addition, the water-soluble nature of this amino acid is also a factor in the increase in its amount during storage. The detection of higher levels of threonine in the control samples of CP with pH 4.00, 5.00 and 6.00 than the raspberry-added samples shows that raspberry powder has a preservative effect on the degradation of protein structure in CP.

As seen in Table 5, all experimental factors and all possible interactions significantly (*p* < 0.01) affected the non-essential amino acids (alanine, arginine, aspartic acid, cystine, glutamic acid, glycine, histidine, ornithine, proline, serine and tyrosine), except for the insignificant (*p* > 0.05) effect of ST on tyrosine content. The amounts of alanine, arginine, glycine, ornithine, proline, serine and tyrosine increased as a result of pH increase (*p <* 0.05) (Table 5). Also, CP with initial pH 4.00 had higher aspartic acid, glutamic acid and histidine contents. Previously, El-Mahdy and El Sabaiy (2007) determined major amino acids in germinating fenugreek seeds as glutamic and aspartic acids. The high amount of glutamic acid in the products used in CP production and in the ERP caused the amount of this amino acid to be high in CP (Table 5). Especially in fenugreek flour, the amount of this amino acid is quite high (Aljuhaimi et al. 2018).

Enhancement CP with various pH by using ERP affected significantly non-essential amino acid contents of samples (*p <* 0.01) (Table 5). Except cystine and ornithine, amounts of these amino acids decreased depending on the amount of ERP. These findings related with quality and stability improvement effects of ERP in CP composition as limiting CP protein degradation. Except for tyrosine (*p* > 0.05), the amounts of other amino acids also changed depending on the storage time (*p* < 0.01). Histidine, ornithine and proline contents increased at the end of the storage (*p* < 0.05). There are changes in free amino acid levels in different curing, addition of additives and processing stages in pastırma production (Soyer et al. 2011; Erdemir and Aksu 2017; Aksu and Erdemir 2022). Soyer et al. (2011) highlighted that amount of free amino acids in pastırma can increase during as a result of CP application. It is also related to the final product salt content, which affects solubility.

## Conclusion

The present study investigated the usability of ERP in pastırma CPs with different pH. ERP had remarkable bioactive content and antioxidant capacity as well as high redness. The obtained findings showed that the use of ERP and different pH levels significantly affected the main quality parameters of CP. Utilization of ERP improved CP visual properties by increasing a* values associated with desired redness, and this was also dependent on the initial pH of CP. Lower initial pH values resulted in higher a* values during the storage period. A decrease in a* values of all CP groups was observed during storage, but redness loss was lower in samples with ERP, especially low pH groups. On the other hand, it was concluded that as the amount of ERP added to the CP increased, the pH change during storage decreased, but the stabilizing effect of ERP was limited for CP with high initial pH. Our results revealed that lowering the initial pH of the CP and incorporating sufficient amount of ERP into the formulation is an effective way to increase and stabilize redness, which is an important parameter for pastırma CP. The major amino acids of the ERP were glutamic acid > alanine > glycine > serine > aspartic acid > proline > lysine > valine, while for commercial fenugreek flour these were glutamic acid > aspartic acid > lysine > leucine > arginine > serine > glycine > alanine. By decreasing the pH value of CP, the amount of essential and non-essential amino acids in CP generally decreased. With the increase of the ERP added to CP, the amount of isoleucine, methionine, and ornithine in CP increased. With storage time, the amounts of leucine, methionine, valine, threonine, histidine, ornithine, and proline increased, the amounts of phenylalanine and tyrosine did not change, and the amounts of other amino acids decreased. However, as the amount of ERP added to the CP composition increased, the change in amino acid amounts decreased. In general, essential and non-essential amino acid contents of CPs decreased with increasing ERP amount and decreasing initial pH. However, the protective effect of ERP on the degradation of proteins in CP during storage was determined. In conclusion, adjusting the initial pH of CP to 3.0–4.0 and incorporating > 4.0% ERP into the formulation may be recommended to increase redness and maintain stability during storage.

## Electronic supplementary material

Below is the link to the electronic supplementary material.

Supplementary Material 1

Supplementary Material 2

## Data Availability

The datasets generated during and/or analysed during the current study are available from the corresponding author on reasonable request.

## Abbreviations

ERP: Encapsulated raspberry powders
CP: Cemen paste
CPs: Cemen pastes
TPC: Total phenolic content
*L**: Lightness
*a**: Redness
*b**: Yellowness
*C**: Chroma
^*o*^*h*: Hue angle
I-pH: Initial pH of cemen paste
ERPL: Encapsulated raspberry powder levels
ST: Storage time
FRAP: Ferric ion reducing antioxidant power
