# Supplementary material for: Properties of encapsulated raspberry powder and its efficacy for improving the color stability and amino acid composition of pastırma cemen pastes with different pH during long term cold-storage
Source: J Food Sci Technol. 2024 Jul 20;62(2):310–24. doi: 10.1007/s13197-024-06029-6 (PMC11757660; doi:10.1007/s13197-024-06029-6)
Supplement: Supplementary file 1 — Supplementary Material 1 [file 13197_2024_6029_MOESM1_ESM.docx]

**Supplementary Material 1.**  Cemen paste samples including various amounts of encapsulated raspberry powder (ERP) at various stages of storage

| **Day 0** | **Day 30** | **Day 60** | **Day 90** | **Day 120** |
| --- | --- | --- | --- | --- |
| **** | **** | **** | **** | **** |
|  | **** | **** | **** | **** |

**Supplementary Material 2.**  Color and pH values of cemen pastes during the storage period (120 days)

|  | **Initial CP**  **pH** | **ERP** | **Storage Time (days)** | | | | |
| --- | --- | --- | --- | --- | --- | --- | --- |
|  |  |  | **0** | **30** | **60** | **90** | **120** |
|  | **3.00** | 0.00% | 48.84±0.22^aAL^ | 46.70±1.08^abAL^ | 45.31±0.54^bcAK^ | 43.37±2.71^cAKL^ | 45.34±1.29^bcAL^ |
|  |  | 3.00% | 44.60±0.37^aBK^ | 44.34±0.53^aBKL^ | 43.23±0.42^aBK^ | 43.42±0.76^aAK^ | 41.40±1.81^bBK^ |
| ***L**** |  | 4.00% | 44.75±0.50^aBM^ | 43.49±1.17^abBL^ | 41.89±0.28^bcCK^ | 41.15±1.60^cAK^ | 42.37±0.19^bcBK^ |
|  |  | 5.00% | 42.74±0.65^aCM^ | 41.43±0.29^bcCM^ | 41.73±0.90^bCK^ | 40.45±0.30^cAK^ | 40.78±0.26^bcBK^ |
|  | **4.00** | 0.00% | 50.83±0.62^aAK^ | 51.09±0.52^aAK^ | 48.16±0.92^bAK^ | 46.62±0.44^cAK^ | 47.64±0.21^bcAK^ |
|  |  | 3.00% | 47.39±0.79^aBK^ | 44.76±0.72^bBK^ | 42.48±2.02^cdBK^ | 42.94±0.72^bcBK^ | 40.74±0.34^dBK^ |
|  |  | 4.00% | 45.06±0.38^aCLM^ | 43.90±0.51^aBL^ | 41.44±1.73^bBK^ | 42.05±0.43^bBK^ | 41.05±0.82^bBL^ |
|  |  | 5.00% | 45.80±0.44^aCK^ | 42.61±0.29^bCL^ | 42.04±0.62^bBK^ | 41.75±0.83^bBK^ | 40.35±0.67^cBK^ |
|  | **5.00** | 0.00% | 48.87±0.67^aAL^ | 46.79±0.58^abAL^ | 45.47±1.30^bAK^ | 42.50±2.62^cAL^ | 42.29±0.53^cAM^ |
|  |  | 3.00% | 46.88±2.65^aAK^ | 44.43±1.94^abBCKL^ | 44.37±1.23^abBK^ | 43.37±0.80^bAK^ | 42.86±0.76^bAK^ |
|  |  | 4.00% | 47.53±1.13^aAK^ | 46.63±1.08^aABK^ | 43.41±0.70^bABK^ | 40.02±2.16^cAK^ | 42.44±0.95^bAK^ |
|  |  | 5.00% | 45.92±0.37^aAK^ | 43.82±0.48^bCK^ | 42.07±1.25^bBK^ | 42.77±1.41^bAK^ | 37.73±0.93^cBL^ |
|  | **6.00** | 0.00% | 45.68±1.31^abAM^ | 48.00±0.64^aAL^ | 45.63±2.90^abAK^ | 43.11±1.01^bAKL^ | 44.98±1.28^abAL^ |
|  |  | 3.00% | 44.43±1.46^aAK^ | 42.41±0.58^aBCL^ | 43.90±0.55^aAK^ | 40.33±1.20^bAL^ | 42.35±1.32^aBK^ |
|  |  | 4.00% | 46.27±0.41^aAKL^ | 42.76±0.29^bBL^ | 42.09±2.98^bAK^ | 41.12±1.09^bAK^ | 42.76±0.31^bBK^ |
|  |  | 5.00% | 44.92±0.20^aAL^ | 41.60±0.43^bCM^ | 41.35±1.11^bAK^ | 42.80±3.33^abAK^ | 41.12±0.52^bBK^ |
| ***a**** | **3.00** | 0.00% | 26.20±0.16^aCK^ | 24.24±0.53^bBK^ | 24.25±0.46^bBK^ | 23.83±0.53^bCK^ | 23.71±0.32^bBK^ |
|  |  | 3.00% | 27.87±0.22^aAK^ | 25.76±0.07^bAK^ | 25.32±0.29^cAK^ | 24.42±0.11^dBK^ | 23.67±0.44^eBK^ |
|  |  | 4.00% | 27.35±0.23^aBK^ | 25.93±0.16^bAK^ | 25.54±0.09^cAK^ | 25.47±0.20^cAK^ | 25.03±0.18^dAK^ |
|  |  | 5.00% | 27.96±0.08^aAK^ | 26.16±0.10^bAK^ | 25.45±0.03^cAK^ | 24.94±0.13^dABK^ | 25.35±0.07^cAK^ |
|  | **4.00** | 0.00% | 20.26±0.05^aCL^ | 17.83±0.21^cBL^ | 17.93±0.39^cBL^ | 18.78±0.27^bCL^ | 17.95±0.15^cCL^ |
|  |  | 3.00% | 24.89±0.19^aBL^ | 23.64±0.36^bAL^ | 23.00±0.40^cAL^ | 23.27±0.25^bcAL^ | 22.81±0.23^cAL^ |
|  |  | 4.00% | 25.22±0.18^aAL^ | 23.75±0.02^bAL^ | 23.41±0.15^cAL^ | 23.06±0.23^dABL^ | 22.76±0.16^dABL^ |
|  |  | 5.00% | 25.19±0.13^aAL^ | 24.70±1.46^aAL^ | 23.43±0.07^bAL^ | 22.78±0.08^bBL^ | 22.44±0.16^bBL^ |
|  | **5.00** | 0.00% | 19.09±0.43^aABM^ | 18.19±0.22^bAL^ | 16.65±0.59^cBM^ | 14.77±0.37^dCM^ | 16.71±0.26^cAM^ |
|  |  | 3.00% | 19.66±0.36^aAM^ | 18.57±0.21^bAM^ | 17.55±0.29^cAM^ | 17.15±0.44^cBM^ | 17.32±0.37^cAM^ |
|  |  | 4.00% | 18.10±0.23^abCM^ | 18.46±0.22^aAM^ | 17.50±0.38^abAM^ | 17.80±0.32^abAM^ | 16.85±1.37^cAM^ |
|  |  | 5.00% | 18.76±0.13^aBM^ | 17.42±0.25^bBM^ | 17.26±0.24^bABM^ | 17.06±0.09^bBM^ | 16.32±0.39^cAM^ |
|  | **6.00** | 0.00% | 18.24±0.67^aAN^ | 14.47±0.35^bCM^ | 13.40±0.37^cAN^ | 13.02±0.23^cCN^ | 11.97±0.20^dCN^ |
|  |  | 3.00% | 18.41±0.19^aAN^ | 15.15±0.26^bBN^ | 14.35±0.81^bAN^ | 13.46±0.38^cBCN^ | 14.42±0.50^bABN^ |
|  |  | 4.00% | 17.33±0.21^aBN^ | 14.90±0.19^bBCN^ | 14.07±1.29^bAN^ | 14.19±0.63^bABN^ | 13.85±0.33^bBN^ |
|  |  | 5.00% | 17.48±0.27^aBN^ | 16.24±0.12^bAM^ | 14.98±0.43^cAN^ | 14.30±0.33^dAN^ | 15.04±0.26^cAN^ |
|  | **3.00** | 0.00% | 43.11±0.25^aAK^ | 41.00±1.33^bAK^ | 37.90±0.52^cAK^ | 38.10±0.95^cAK^ | 38.76±0.78^cAK^ |
|  |  | 3.00% | 39.53±0.38^aBL^ | 37.94±0.24^bBK^ | 36.91±0.37^bcBK^ | 35.81±1.49^cdABK^ | 35.26±0.71^dBK^ |
| ***b**** |  | 4.00% | 38.66±0.90^aBL^ | 37.68±0.66^abBK^ | 36.46±0.52^bcBCK^ | 36.99±1.75^abcABK^ | 35.37±0.22^cBK^ |
|  |  | 5.00% | 38.56±0.45^aBL^ | 37.23±0.27^bBK^ | 35.60±0.41^cCK^ | 35.15±0.87^cdBK^ | 34.47±0.25^dBK^ |
|  | **4.00** | 0.00% | 40.49±0.32^aAL^ | 37.08±0.16^bBL^ | 35.19±0.78^cdBL^ | 36.03±0.89^bcAL^ | 34.22±0.75^dAL^ |
|  |  | 3.00% | 40.98±0.35^aAK^ | 38.51±0.92^bAK^ | 36.02±0.29^cBK^ | 36.46±0.24^cAK^ | 34.57±1.38^dAK^ |
|  |  | 4.00% | 41.28±0.27^aAK^ | 38.02±0.44^bABK^ | 37.94±1.48^bAK^ | 36.14±0.58^cAKL^ | 34.79±0.21^cAK^ |
|  |  | 5.00% | 39.97±0.43^aAK^ | 37.57±0.89^bABK^ | 36.10±0.26^bcBK^ | 35.80±0.90^cAK^ | 33.27±1.27^dAK^ |
|  | **5.00** | 0.00% | 36.57±0.90^aAM^ | 35.13±0.53^aABM^ | 31.75±1.28^bAM^ | 28.31±1.58^cBM^ | 30.99±0.35^bABM^ |
|  |  | 3.00% | 35.26±0.57^aBM^ | 34.23±0.59^aBL^ | 32.76±0.63^bAL^ | 31.39±0.83^cAL^ | 31.88±0.18^bcAL^ |
|  |  | 4.00% | 34.12±0.40^abCM^ | 35.75±0.62^aAL^ | 32.89±0.41^bcAL^ | 33.63±1.87^abcAL^ | 31.05±2.27^cABL^ |
|  |  | 5.00% | 33.13±0.13^aCM^ | 32.39±0.67^abCL^ | 31.26±0.96^bcAL^ | 31.08±0.52^cAL^ | 28.73±0.62^dBL^ |
|  | **6.00** | 0.00% | 33.33±1.45^aAN^ | 31.59±0.27^aAN^ | 31.40±1.04^aAM^ | 26.72±0.67^cBM^ | 29.22±1.30^bAN^ |
|  |  | 3.00% | 33.17±0.48^aAN^ | 28.46±1.01^bBM^ | 28.61±2.15^bAM^ | 27.52±0.62^bBM^ | 29.31±0.75^bAM^ |
|  |  | 4.00% | 32.04±0.36^aAN^ | 29.36±0.14^bBM^ | 28.40±1.94^bAM^ | 27.76±1.67^bABM^ | 29.72±0.23^bAL^ |
|  |  | 5.00% | 29.08±0.30^aBN^ | 28.98±0.42^aBM^ | 28.62±0.49^aAM^ | 29.58±0.77^aAM^ | 29.56±0.95^aAL^ |
| ***C**** | **3.00** | 0.00% | 50.45±0.30^aAK^ | 47.54±1.42^bAK^ | 45.00±0.66^cAK^ | 44.94±1.00^cAK^ | 45.44±0.72^cAK^ |
|  |  | 3.00% | 48.36±0.43^aBK^ | 45.86±0.19^bBK^ | 44.76±0.44^cBK^ | 43.75±0.71^dAK^ | 42.47±0.62^eBK^ |
|  |  | 4.00% | 46.15±1.49^aCL^ | 45.75±0.52^aBK^ | 44.51±0.45^abABK^ | 44.91±1.54^abAK^ | 43.34±0.28^bBK^ |
|  |  | 5.00% | 47.63±0.38^aBCK^ | 45.50±0.18^bBK^ | 43.77±0.35^cBK^ | 43.47±0.11^cAK^ | 42.79±0.16^dBK^ |
|  | **4.00** | 0.00% | 45.28±0.28^aCL^ | 41.15±0.22^bBL^ | 39.49±0.85^cdCL^ | 40.63±0.90^bcBL^ | 38.65±0.66^dBL^ |
|  |  | 3.00% | 47.89±0.23^aAK^ | 45.19±0.96^bAK^ | 42.74±0.44^cdBK^ | 43.25±0.26^cAK^ | 41.42±1.24^dAK^ |
|  |  | 4.00% | 48.37±0.32^aAK^ | 44.84±0.39^bAL^ | 44.58±1.31^bAK^ | 42.87±0.61^cAK^ | 41.58±0.14^dAK^ |
|  |  | 5.00% | 47.24±0.43^aBK^ | 44.45±0.85^bAK^ | 43.04±0.25^cABK^ | 42.43±0.79^cAK^ | 40.14±1.05^dABL^ |
|  | **5.00** | 0.00% | 41.25±0.99^aAM^ | 39.56±0.57^aABM^ | 35.85±1.40^bAM^ | 31.94±1.54^cCM^ | 35.21±0.39^bABM^ |
|  |  | 3.00% | 40.40±0.74^aAL^ | 38.94±0.61^bBL^ | 37.17±0.69^cAL^ | 35.79±0.90^dABL^ | 36.28±0.31^cdAL^ |
|  |  | 4.00% | 38.63±0.36^aBM^ | 40.24±0.65^aAM^ | 37.26±0.54^abAL^ | 38.05±1.81^abAL^ | 35.33±2.79^bABL^ |
|  |  | 5.00% | 38.07±0.15^aBL^ | 36.83±0.69^abCL^ | 35.71±0.92^bAL^ | 35.46±0.49^bBL^ | 32.61±1.45^cBM^ |
|  | **6.00** | 0.00% | 37.99±1.60^aAN^ | 34.75±0.38^bAN^ | 34.14±1.11^bAM^ | 29.72±0.64^cBN^ | 31.57±1.26^cAN^ |
|  |  | 3.00% | 37.94±0.51^aAM^ | 32.24±1.02^bBM^ | 32.01±2.29^bAM^ | 30.57±0.67^bBM^ | 32.23±0.38^bAM^ |
|  |  | 4.00% | 36.85±0.84^aAN^ | 32.92±0.20^bBN^ | 31.70±2.31^bAM^ | 31.17±1.77^bABM^ | 32.37±0.67^bAM^ |
|  |  | 5.00% | 33.93±0.39^aBM^ | 33.21±0.41^abBM^ | 32.28±0.57^bAM^ | 32.86±0.83^abAM^ | 33.16±0.93^abAM^ |
| **^o^*h*** | **3.00** | 0.00% | 58.71±0.02^abAM^ | 59.32±0.33^aAN^ | 57.38±0.28^cAN^ | 57.98±0.54^bcAM^ | 59.40±1.22^aAM^ |
|  |  | 3.00% | 54.82±0.10^bBM^ | 55.83±0.20^aBM^ | 55.55±0.21^aBN^ | 56.06±0.51^aBN^ | 56.12±0.49^aBM^ |
|  |  | 4.00% | 53.73±1.25^bBM^ | 55.45±0.54^aBCM^ | 55.00±0.36^abCN^ | 55.42±1.09^aBM^ | 54.71±0.06^abCN^ |
|  |  | 5.00% | 54.05±0.30^cdBL^ | 54.90±0.28^abCN^ | 54.45±0.29^bcDN^ | 54.99±0.17^aBN^ | 53.66±0.27^dCN^ |
|  | **4.00** | 0.00% | 63.38±0.17^bAK^ | 64.31±0.21^aAL^ | 63.00±0.32^bcAL^ | 62.47±0.32^cdAL^ | 62.32±0.57^dAL^ |
|  |  | 3.00% | 58.69±0.25^aBL^ | 58.45±0.28^aBL^ | 57.43±0.30^bBCM^ | 57.45±0.29^bBM^ | 56.57±0.89^bBM^ |
|  |  | 4.00% | 58.58±0.08^aBL^ | 58.00±0.28^abBCL^ | 58.29±0.92^abBM^ | 57.46±0.16^bcBL^ | 56.80±0.31^cBM^ |
|  |  | 5.00% | 57.78±0.15^aCK^ | 57.69±0.43^aCM^ | 57.00±0.13^abCM^ | 57.52±0.58^aBM^ | 55.97±1.05^bBM^ |
|  | **5.00** | 0.00% | 61.49±1.75^aAL^ | 62.62±0.08^aAM^ | 62.31±0.18^aAM^ | 62.41±0.96^aAL^ | 61.66±0.31^aAL^ |
|  |  | 3.00% | 60.87±0.06^cAK^ | 61.53±0.15^abBK^ | 61.81±0.12^aAL^ | 61.27±0.26^bcAL^ | 61.49±0.43^abAL^ |
|  |  | 4.00% | 62.05±0.43^abAK^ | 62.69±0.12^aAK^ | 61.98±0.27^abAL^ | 62.06±0.85^abAK^ | 61.53±0.57^bAL^ |
|  |  | 5.00% | 59.12±2.31^bAK^ | 61.73±0.14^aBK^ | 61.09±0.57^abBL^ | 61.23±0.34^aAL^ | 60.40±0.13^abBL^ |
|  | **6.00** | 0.00% | 61.31±0.17^eAL^ | 65.38±0.35^cAK^ | 66.89±0.11^bAK^ | 64.04±0.59^dAK^ | 67.71±0.66^aAK^ |
|  |  | 3.00% | 60.97±0.12^dABK^ | 61.97±0.43^cCK^ | 63.35±0.50^bBK^ | 64.17±0.09^aAK^ | 63.81±0.68^abCK^ |
|  |  | 4.00% | 60.37±2.11^cABKL^ | 63.09±0.23^abBK^ | 63.67±0.55^abBK^ | 62.92±0.38^bBK^ | 65.03±0.49^aBK^ |
|  |  | 5.00% | 58.97±0.19^dBK^ | 60.70±0.17^cDL^ | 62.35±0.59^bCK^ | 64.20±0.06^aAK^ | 63.02±0.56^bCK^ |
|  | **3.00** | 0.00% | 3.06±0.02^bBN^ | 2.98±0.01^cAN^ | 3.06±0.02^bAN^ | 3.12±0.02^aCN^ | 3.13±0.01^aBN^ |
|  |  | 3.00% | 3.07±0.02^bBN^ | 3.02±0.02^cAN^ | 3.05±0.01^bcAN^ | 3.16±0.03^aBCM^ | 3.14±0.05^aBN^ |
| **pH** |  | 4.00% | 3.13±0.02^bAN^ | 3.01±0.03^cAN^ | 3.08±0.01^bAN^ | 3.18±0.03^aBN^ | 3.08±0.03^bBN^ |
|  |  | 5.00% | 3.13±0.01^bAN^ | 2.92±0.01^dBN^ | 3.05±0.05^cAN^ | 3.23±0.02^aAN^ | 3.22±0.02^aAN^ |
|  | **4.00** | 0.00% | 4.05±0.01^bAM^ | 4.00±0.02^cAM^ | 4.03±0.01^bAM^ | 4.04±0.01^bAM^ | 4.09±0.02^aAM^ |
|  |  | 3.00% | 4.02±0.03^abABM^ | 3.94±0.03^cBCM^ | 3.98±0.02^bBM^ | 4.03±0.03^aAL^ | 4.03±0.01^aBM^ |
|  |  | 4.00% | 3.99±0.04^abBM^ | 3.90±0.01^cCM^ | 3.96±0.03^bBM^ | 4.00±0.02^abBM^ | 4.03±0.02^aBM^ |
|  |  | 5.00% | 4.05±0.02^aAM^ | 3.96±0.03^bABM^ | 3.96±0.01^bBM^ | 3.99±0.01^bBM^ | 4.03±0.01^aBM^ |
|  | **5.00** | 0.00% | 5.03±0.02^aAL^ | 5.00±0.01^bAL^ | 5.05±0.02^aAK^ | 5.05±0.01^aAK^ | 5.06±0.01^aAK^ |
|  |  | 3.00% | 4.92±0.02^bcBL^ | 4.91±0.02^cBL^ | 4.96±0.01^abBL^ | 4.98±0.04^aBK^ | 4.96±0.01^abBK^ |
|  |  | 4.00% | 4.86±0.01^aCL^ | 4.85±0.01^aCL^ | 4.85±0.01^aCL^ | 4.85±0.01^aCL^ | 4.85±0.01^aCK^ |
|  |  | 5.00% | 4.78±0.01^cDL^ | 4.76±0.01^cDL^ | 4.82±0.01^bDL^ | 4.86±0.02^aCL^ | 4.84±0.02^aCK^ |
|  | **6.00** | 0.00% | 6.01±0.03^aAK^ | 5.89±0.03^bAK^ | 4.85±0.01^cDL^ | 4.59±0.02^dDL^ | 4.56±0.08^dBL^ |
|  |  | 3.00% | 5.70±0.01^aBK^ | 5.64±0.02^aBK^ | 5.25±0.05^bCK^ | 4.95±0.04^cCK^ | 4.83±0.04^dAL^ |
|  |  | 4.00% | 5.59±0.01^aCK^ | 5.55±0.01^bCK^ | 5.50±0.01^cAK^ | 5.21±0.02^dAK^ | 4.79±0.02^eAL^ |
|  |  | 5.00% | 5.48±0.01^aDK^ | 5.46±0.03^aDK^ | 5.37±0.06^bBK^ | 5.10±0.09^cBK^ | 4.80±0.02^dAL^ |

Mean ± Standard Deviation. ERP; encapsulated raspberry powder. Different lowercase letters (a-e) in the same row indicate significant differences between storage days for the same treatment (P<0.05). Different uppercase letters (A-D) in the same column indicate significant differences between between ERP treatments for the same storage day (P<0.05). Different uppercase letters (K-N) in the same column indicate significant differences between the initial cemen pH values for the same ERP treatment (P<0.05).

**Supplementary Material 3.**  Amino acid contents of cemen paste samples including various amounts of encapsulated raspberry powder (ERP) at various stages of storage

| Amino  Acids | ST | pH 3.0 | | | | pH 4.0 | | | | pH 5.0 | | | | pH 6.0 | | | |
| --- | --- | --- | --- | --- | --- | --- | --- | --- | --- | --- | --- | --- | --- | --- | --- | --- | --- |
|  |  | Encapsulated Raspberry Powder Levels (ERPL) | | | | | | | | | | | | | | | |
|  |  | 0% | 3% | 4% | 5% | 0% | 3% | 4% | 5% | 0% | 3% | 4% | 5% | 0% | 3% | 4% | 5% |
| Essential Amino Acids | | | | | | | | | | | | | | | | | |
| Lysine | 0 | 1546.97±6.85^bAL^ | 1624.82  ±7.93^aAM^ | 1270.51  ±8.36^dAM^ | 1334.52  ±11.32^cAN^ | 1534.25  ±7.92^aBL^ | 1553.57  ±6.27^aAN^ | 1406.75  ±4.12^bAL^ | 1552.92  ±11.21^aAL^ | 1444.50  ±7.09^cBM^ | 1644.95  ±3.63^bAL^ | 1396.94  ±2.34^cBL^ | 1694.95  ±34.96^aAK^ | 1590.04  ±3.12^bAK^ | 1667.96  ±2.79^aAK^ | 1457.07  ±3.66^dAK^ | 1476.11  ±3.99^cAM^ |
|  | 120 | 673.35  ±0.21^dBN^ | 1174.82  ±0.75^bBL^ | 1196.99  ±0.76^aBN^ | 920.60  ±0.56^cBN^ | 1830.13  ±0.67^aAK^ | 1097.96  ±1.85^dBM^ | 1247.40  ±0.86^bBM^ | 1207.37  ±0.37^cBM^ | 1665.62  ±0.14^aAL^ | 1050.89  ±0.72^dBN^ | 1406.70  ±1.61^cAK^ | 1549.29  ±3.06^bBK^ | 1445.44  ±2.50^bBM^ | 1566.20  ±0.88^aBK^ | 1306.17  ±0.35^dBL^ | 1324.21  ±0.58^cBL^ |
| Isoleucine | 0 | 451.79  ±0.86^bcAM^ | 519.55  ±1.32^aAM^ | 432.61  ±18.31^cAM^ | 473.37  ±3.42^bAM^ | 524.49  ±0.60^bBK^ | 507.47  ±0.37^cAN^ | 527.68  ±9.04^bAK^ | 556.61  ±3.35^aAK^ | 437.25  ±1.18^dBN^ | 547.10  ±0.83^bAL^ | 456.97  ±0.64^cBLM^ | 564.76  ±11.05^aBK^ | 509.17  ±0.01^cBL^ | 585.18  ±0.70^aBK^ | 467.54  ±1.84^dBL^ | 532.14  ±1.04^bAL^ |
|  | 120 | 111.80  ±0.20^dBN^ | 422.81  ±0.49^bBL^ | 473.78  ±0.30^aAM^ | 346.26  ±0.86^cBN^ | 660.83  ±0.79^aAK^ | 367.76  ±0.68^dBM^ | 459.86  ±0.38^bBN^ | 417.39  ±4.29^cBM^ | 624.79  ±0.33^bAL^ | 134.92  ±0.35^dBN^ | 542.71  ±0.62^cAK^ | 658.20  ±3.50^aAK^ | 615.21  ±0.13^bAM^ | 695.15  ±0.83^aAK^ | 518.45  ±3.77^cAL^ | 520.83  ±2.07^cBL^ |
| Leucine | 0 | 1150.71  ±5.54^bAL^ | 1242.73  ±59.42^aAKL^ | 998.18  ±11.90^cBL^ | 1045.17  ±11.99^cAM^ | 1146.69  ±36.16^bBL^ | 1170.69  ±16.96^bAL^ | 1189.52  ±76.22^bAK^ | 1329.88  ±9.30^aAK^ | 992.78  ±4.79^dBM^ | 1317.74  ±3.83^bAK^ | 1035.23  ±1.61^cBL^ | 1348.43  ±10.74^aBK^ | 1341.38  ±1.41^aBK^ | 1322.01  ±20.75^aBK^ | 1094.48  ±45.10^bBKL^ | 1163.54  ±20.83^bAL^ |
|  | 120 | 834.39  ±18.92^dBN^ | 1254.66  ±26.48^aAL^ | 1189.26  ±2.07^bAN^ | 978.85  ±4.41^cBN^ | 1926.58  ±0.45^aAK^ | 924.18  ±1.25^dBM^ | 1360.71  ±0.47^bAK^ | 1165.83  ±0.86^cBL^ | 1552.95  ±3.23^aAL^ | 725.78  ±0.74^dBN^ | 1298.33  ±1.32^cAL^ | 1508.53  ±0.40^bAK^ | 1504.94  ±9.38^bAM^ | 1576.87  ±9.37^aAK^ | 1285.37  ±5.57^cAM^ | 1100.65  ±0.60^dAM^ |
| Methionine | 0 | 105.30  ±0.27^cBM^ | 132.40  ±0.77^aBL^ | 101.59  ±0.70^dBM^ | 124.91  ±0.13^bBL^ | 115.49  ±0.55^cBK^ | 139.29  ±0.53^aAK^ | 139.32  ±0.46^aBK^ | 135.24  ±1.25^bAK^ | 70.97  ±0.45^cBN^ | 104.80  ±0.28^bAN^ | 104.95  ±0.12^bBL^ | 119.69  ±2.35^aBM^ | 112.11  ±0.28^aBL^ | 109.54  ±0.61^bBM^ | 82.35  ±0.69^cBN^ | 68.43  ±0.21^dBN^ |
|  | 120 | 111.62  ±0.45^dAN^ | 172.52  ±0.32^aAL^ | 133.86  ±0.06^cAM^ | 141.85  ±0.21^bAN^ | 241.12  ±0.21^aAK^ | 127.34  ±0.02^dBM^ | 192.48  ±1.64^bAL^ | 177.61  ±0.04^cBM^ | 191.47  ±0.45^cAL^ | 103.66  ±0.02^dBN^ | 197.41  ±0.83^aAK^ | 195.34  ±0.28^bAL^ | 165.76  ±0.05^cAM^ | 236.72  ±0.09^aAK^ | 192.63  ±1.34^bAL^ | 238.20  ±0.21^aAK^ |
| Phenylalanine | 0 | 932.60  ±3.22^bAM^ | 1028.01  ±7.96^aAM^ | 841.04  ±1.82^cBL^ | 904.05  ±19.76^bAM^ | 1021.52  ±9.20^bBL^ | 1003.22  ±4.73^bcAN^ | 971.28  ±7.55^cAK^ | 1131.16  ±20.41^aAK^ | 907.97  ±5.70^dBN^ | 1069.91  ±1.32^aAL^ | 974.78  ±5.78^cBK^ | 1045.60  ±0.13^bBL^ | 1064.63  ±9.29^bAK^ | 1171.26  ±0.73^aBK^ | 984.72  ±18.05^cAK^ | 974.36  ±42.53^cAM^ |
|  | 120 | 708.67  ±66.85^bBN^ | 941.70  ±2.16^aBL^ | 891.52  ±0.50^aAN^ | 918.33  ±13.99^aAM^ | 1377.49  ±4.11^aAK^ | 863.13  ±0.15^dBM^ | 953.38  ±2.65^cAM^ | 969.90  ±0.56^bBL^ | 1172.82  ±0.49^aAL^ | 744.75  ±0.58^dBN^ | 1057.19  ±2.14^bAK^ | 1051.71  ±1.76^cAK^ | 1069.53  ±3.18^bAM^ | 1230.21  ±5.00^aAK^ | 995.90  ±0.47^cAL^ | 963.45  ±4.24^dAL^ |
| Valine | 0 | 824.30  ±2.77^bAM^ | 896.97  ±5.08^aAM^ | 778.25  ±6.15^cBM^ | 833.49  ±8.16^bAM^ | 887.24  ±3.71^cBL^ | 927.74  ±2.90^bAL^ | 869.15  ±2.58^dBL^ | 983.75  ±6.66^aAK^ | 822.64  ±4.02^cBM^ | 993.40  ±2.64^aAK^ | 879.23  ±0.44^bBL^ | 967.51  ±18.39^aBK^ | 920.73  ±1.93^bBK^ | 985.91  ±0.81^aBK^ | 892.44  ±3.42^dBK^ | 902.66  ±1.38^cBL^ |
|  | 120 | 549.99  ±0.15^dBN^ | 876.19  ±0.88^aBL^ | 852.63  ±2.77^bAN^ | 811.00  ±0.04^cAN^ | 1298.09  ±0.79^aAK^ | 851.78  ±1.91^dBM^ | 968.99  ±0.05^bAM^ | 876.25  ±3.69^cBM^ | 1182.37  ±0.47^bAM^ | 638.15  ±0.14^dBN^ | 1156.01  ±0.25^cAK^ | 1239.32  ±0.39^aAK^ | 1188.81  ±2.93^bAL^ | 1218.37  ±0.63^aAK^ | 1077.24  ±0.57^cAL^ | 1054.28  ±0.16^dAL^ |
| Threonine | 0 | 496.55  ±0.66^bBL^ | 520.91  ±3.92^aBN^ | 458.02  ±11.04^cBK^ | 441.04  ±2.32^dBN^ | 441.58  ±1.92^cBN^ | 595.34  ±2.22^bBL^ | 393.28  ±1.90^dBL^ | 623.11  ±4.62^aBK^ | 462.85  ±2.63^cBM^ | 562.69  ±1.81^aBM^ | 463.09  ±0.34^cBK^ | 516.91  ±9.66^bBL^ | 578.04  ±1.38^bBK^ | 619.38  ±0.81^aBK^ | 397.73  ±1.36^dBL^ | 485.07  ±2.30^cBM^ |
|  | 120 | 701.19  ±11.78^dAN^ | 1063.48  ±1.45^aAL^ | 997.99  ±0.28^bAM^ | 955.72  ±0.52^cAM^ | 1436.70  ±2.31^aAK^ | 1002.26  ±1.13^dAM^ | 1023.24  ±1.55^cAL^ | 1096.62  ±3.69^bAL^ | 1238.17  ±1.28^aAL^ | 729.92  ±0.48^dAN^ | 1216.44  ±5.03^bAK^ | 1142.03  ±3.24^cAK^ | 1206.73  ±0.41^aAM^ | 1173.12  ±0.35^bAK^ | 946.79  ±1.06^cAN^ | 924.95  ±0.64^dAN^ |
| Non-essential Amino Acids | | | | | | | | | | | | | | | | | |
| Aspartic  Acid | 0 | 2566.04  ±12.95^aAK^ | 2596.39  ±16.05^aAL^ | 2456.26  ±16.00^bAL^ | 2316.56  ±20.54^cAM^ | 2431.82  ±15.86^dBM^ | 2479.43  ±8.93^cAM^ | 2983.70  ±11.94^aAK^ | 2830.27  ±20.25^bAK^ | 2255.98  ±10.98^cBN^ | 2472.17  ±0.26^bAM^ | 2201.30  ±0.69^cAN^ | 2715.12  ±52.28^aAL^ | 2481.38  ±5.81^bAL^ | 3004.60  ±3.18^aAK^ | 2477.22  ±19.39^bAL^ | 2310.14  ±9.09^cAM^ |
|  | 120 | 1855.57  ±1.99^dBN^ | 2477.32  ±2.84^aBL^ | 2255.51  ±1.48^bBL^ | 2029.46  ±0.68^cBN^ | 3603.09  ±9.87^aAK^ | 1978.13  ±2.60^dBM^ | 2421.33  ±6.97^bBK^ | 2373.01  ±3.08^cBL^ | 2831.04  ±5.76^aAL^ | 1815.15  ±1.86^dBN^ | 1913.54  ±2.97^cBN^ | 2483.17  ±2.24^bBK^ | 2239.54  ±4.55^cBM^ | 2722.84  ±0.39^aBK^ | 2138.41  ±9.62^dBM^ | 2340.07  ±13.54^bAM^ |
| Cystine | 0 | 454.76  ±0.40^cAN^ | 500.35  ±2.91^aAM^ | 466.71  ±3.25^bAM^ | 457.30  ±4.17^cAL^ | 499.60  ±1.88^aAK^ | 471.59  ±1.41^bAN^ | 497.85  ±1.69^aAL^ | 471.13  ±3.34^bAL^ | 476.17  ±2.81^cAL^ | 513.66  ±1.18^aAL^ | 520.12  ±0.40^aAK^ | 496.96  ±10.86^bAK^ | 463.46  ±0.84^cAM^ | 550.18  ±1.41^aAK^ | 470.42  ±0.62^bAM^ | 457.18  ±3.35^dAL^ |
|  | 120 | 340.04  ±0.93^dBN^ | 434.66  ±1.29^aBL^ | 347.48  ±0.51^cBL^ | 357.87±0.04^bBM^ | 449.91  ±7.021^aBK^ | 435.14  ±0.63^abBL^ | 368.84  ±23.51^cBL^ | 414.31  ±1.73^bBL^ | 363.57  ±6.48^cBM^ | 439.77  ±0.83^aBK^ | 402.84  ±3.95^bBK^ | 445.49  ±1.78^aBK^ | 378.87  ±0.36^aBL^ | 359.91  ±2.89^bBM^ | 363.92  ±1.13^bBL^ | 354.98  ±0.23^cBM^ |
| Arginine | 0 | 1822.04  ±7.74^aAK^ | 1790.87  ±9.62^bAN^ | 1581.70  ±11.72^cAN^ | 1587.85  ±14.52^cAL^ | 1797.73  ±8.29^cBL^ | 1825.31  ±9.04^bAM^ | 1759.70  ±5.07^dAK^ | 1933.33  ±13.46^aAK^ | 1710.25  ±11.75^cBM^ | 2026.63  ±2.48^aAL^ | 1686.77  ±1.12^cBL^ | 1914.30  ±39.02^bAK^ | 1810.91  ±2.83^cBKL^ | 2049.98  ±2.32^aBK^ | 1664.21  ±1.36^dBM^ | 1874.34  ±5.43^bAK^ |
|  | 120 | 1151.66  ±0.60^dBN^ | 1656.96  ±0.88^aBL^ | 1480.90  ±0.74^cBN^ | 1493.43  ±1.20^bBN^ | 2653.16  ±2.74^aAK^ | 1469.57  ±0.38^dBN^ | 1732.90  ±1.37^bBM^ | 1606.82  ±0.55^cBL^ | 2139.12  ±0.70^aAL^ | 1531.87  ±0.84^dBM^ | 1849.90  ±0.91^cAK^ | 1913.85  ±2.87^bAK^ | 1935.53  ±1.86^bAM^ | 2149.15  ±0.71^aAK^ | 1779.76  ±0.12^cAL^ | 1600.02  ±1.14^dBM^ |
| Glutamic Acid | 0 | 3941.64  ±14.77^bAL^ | 4085.22  ±35.18^aAM^ | 3409.51  ±25.26^cAN^ | 3378.75  ±28.89^cAN^ | 3880.52  ±15.78^cBM^ | 3974.46  ±1.79^bAN^ | 3878.29  ±14.26^cAK^ | 4203.35  ±29.90^aAK^ | 3687.59  ±21.56^cBN^ | 4227.93  ±16.96^aAL^ | 3771.19  ±2.93^cBL^ | 4006.32  ±85.03^bAL^ | 4229.62  ±1.36^bAK^ | 4529.73  ±9.03^aAK^ | 3592.76  ±4.55^dBM^ | 3812.17  ±11.75^cAM^ |
|  | 120 | 2655.29  ±1.66^dBN^ | 3635.81  ±3.90^aBL^ | 3469.50  ±1.03^bAN^ | 3315.96  ±1.08^cAN^ | 5339.76  ±10.72^aAK^ | 3559.44  ±0.62^dBM^ | 3917.68  ±5.63^bAK^ | 3846.11  ±1.35^cBL^ | 4847.10  ±9.86^aAL^ | 2946.40  ±0.23^dBN^ | 3856.14  ±1.04^cAL^ | 4179.98  ±4.44^bAK^ | 3803.13  ±4.87^bBM^ | 4501.40  ±6.35^aAK^ | 3613.03  ±1.16^dAM^ | 3657.94  ±1.86^cBM^ |
| Ornithine | 0 | 227.06  ±0.65^bBN^ | 221.68  ±1.24^cBN^ | 181.20  ±1.30^dBN^ | 236.24  ±2.14^aBM^ | 253.17  ±0.85^aBL^ | 226.65  ±1.57^cBM^ | 251.59  ±0.77^aBL^ | 246.44  ±1.90^bBL^ | 230.71  ±1.26^bBM^ | 290.24  ±0.59^aBK^ | 227.71  ±0.11^bBM^ | 230.48  ±6.48^bBM^ | 259.09  ±0.76^bBK^ | 232.77  ±0.33^dBL^ | 281.79  ±0.24^aBK^ | 257.58  ±0.60^cBK^ |
|  | 120 | 392.61  ±1.45^dAK^ | 410.46  ±0.13^bAK^ | 446.84  ±0.77^aAK^ | 400.24  ±0.28^cAL^ | 362.04  ±0.39^cAL^ | 325.46  ±0.33^dAM^ | 413.64  ±1.22^bAL^ | 463.55  ±0.21^aAK^ | 349.02  ±1.02^cAM^ | 400.81  ±0.33^aAL^ | 334.61  ±1.09^dAN^ | 367.52  ±4.38^bAM^ | 350.80  ±0.21^dAM^ | 400.15  ±0.76^bAL^ | 395.12  ±0.34^cAM^ | 405.28  ±0.76^aAL^ |
| Glycine | 0 | 1947.61  ±33.06^aAM^ | 1910.43  ±7.33^aAL^ | 1676.82  ±16.1^bAM^ | 1597.93  ±12.41^cAM^ | 2013.05  ±2.49^aAL^ | 1882.83  ±0.79^cAL^ | 1700.11  ±5.23^dAM^ | 1958.79  ±11.07^bAL^ | 1916.96  ±11.24^bAM^ | 2056.16  ±16.09^aAK^ | 1773.45  ±0.57^cAL^ | 1910.27  ±36.19^bAL^ | 2181.91  ±28.37^aAK^ | 1798.37  ±33.79^cAM^ | 1851.42  ±2.62^cAK^ | 2089.06  ±8.45^bAK^ |
|  | 120 | 691.77  ±3.47^dBN^ | 1041.51  ±2.44^bBM^ | 1294.43  ±0.54^aBK^ | 785.62  ±0.83^cBN^ | 1655.39  ±0.99^aBK^ | 1009.23  ±4.19^dBN^ | 1058.03  ±1.79^cBM^ | 1132.17  ±1.17^bBK^ | 1614.49  ±2.73^aBL^ | 1112.44  ±16.62^bBL^ | 902.82  ±0.96^cBN^ | 922.82  ±2.74^cBM^ | 972.08  ±0.35^dBM^ | 1398.11  ±6.37^aBK^ | 1156.86  ±0.67^bBL^ | 1083.00  ±4.31^cBL^ |
| Proline | 0 | 1166.41  ±4.40^bAM^ | 1190.72  ±7.20^aBM^ | 1044.60  ±7.38^dBM^ | 1089.02  ±9.45^cBL^ | 1193.25  ±4.34^bBL^ | 1181.58  ±5.76^bAM^ | 1181.71  ±5.01^bBK^ | 1228.36  ±8.55^aBK^ | 1164.16  ±6.46^cBM^ | 1303.98  ±4.72^aAL^ | 1161.82  ±0.07^cBL^ | 1236.78  ±24.43^bBK^ | 1272.89  ±3.17^bBK^ | 1320.32  ±2.84^aBK^ | 1152.14  ±0.79^dBL^ | 1225.00  ±4.24^cAK^ |
|  | 120 | 973.56  ±0.42^dBN^ | 1225.05  ±0.41^aAL^ | 1172.61  ±0.67^bAN^ | 1152.74  ±0.09^cAN^ | 1656.98  ±2.34^aAK^ | 1173.34  ±0.35^dAM^ | 1320.49  ±0.52^bAL^ | 1257.76  ±0.50^cAL^ | 1439.07  ±0.43^aAL^ | 1116.45  ±0.22^dBN^ | 1341.17  ±0.04^cAK^ | 1408.99  ±1.08^bAK^ | 1394.30  ±0.44^bAM^ | 1485.67  ±0.27^aAK^ | 1251.03  ±1.25^cAM^ | 1192.78  ±0.58^dBM^ |
| Serine | 0 | 1454.47  ±4.84^aAM^ | 1451.94  ±9.47^aAN^ | 1324.23  ±8.88^cAN^ | 1423.53  ±11.61^bAL^ | 1504.46  ±5.03^aBL^ | 1504.09  ±5.64^aAM^ | 1446.73  ±4.46^bAM^ | 1428.45  ±10.13^bAL^ | 1436.61  ±8.60^cAN^ | 1606.15  ±5.13^aAL^ | 1520.37  ±0.67^bAK^ | 1567.98  ±31.21^aAK^ | 1609.65  ±4.36^bAK^ | 1771.18  ±3.54^aAK^ | 1485.07  ±0.09^dAL^ | 1548.46  ±3.77^cAK^ |
|  | 120 | 1042.59  ±1.88^dBN^ | 1307.39  ±2.28^aBK^ | 1170.76  ±0.98^cBN^ | 1238.12  ±0.37^bBL^ | 1713.07  ±1.74^aAK^ | 1192.26  ±0.88^dBM^ | 1365.94  ±2.51^bBK^ | 1214.09  ±3.48^cBM^ | 1438.31  ±4.25^aAM^ | 1089.85  ±3.03^dBN^ | 1311.36  ±1.24^bBL^ | 1299.51  ±0.28^cBK^ | 1513.29  ±0.79^aBL^ | 1295.04  ±1.39^bBL^ | 1294.46  ±0.05^bBM^ | 1130.30  ±1.00^cBN^ |
| Alanine | 0 | 1348.60  ±3.95^aAN^ | 1336.53  ±2.96^aAN^ | 1276.75  ±10.23^cAM^ | 1309.29  ±9.98^bAM^ | 1414.23  ±5.38^bAL^ | 1404.33  ±7.08^bAM^ | 1396.90  ±4.23^bAL^ | 1505.28  ±11.19^aAK^ | 1387.24  ±7.69^bAM^ | 1452.48  ±2.02^aAL^ | 1447.18  ±0.47^aAK^ | 1468.56  ±28.67^aAK^ | 1523.32  ±3.13^bAK^ | 1611.76  ±3.02^aAK^ | 1401.04  ±1.00^dAL^ | 1419.73  ±4.12^cAL^ |
|  | 120 | 371.47  ±0.38^dBN^ | 662.74  ±0.88^aBL^ | 565.04  ±0.92^cBN^ | 648.57  ±0.49^bBL^ | 908.05  ±0.57^aBK^ | 517.71  ±0.26^cBN^ | 690.77  ±0.75^bBM^ | 496.44  ±0.04^dBN^ | 828.65  ±1.61^aBL^ | 619.94  ±0.24^dBM^ | 814.21  ±0.71^bBK^ | 715.93  ±0.11^cBK^ | 793.77  ±0.09^bBM^ | 871.49  ±2.58^aBK^ | 695.88  ±1.34^cBL^ | 640.57  ±1.29^dBM^ |
| Tyrosine | 0 | 531.39  ±2.79^bAL^ | 593.46  ±3.16^aAM^ | 410.63  ±2.90^dBM^ | 488.30  ±0.64^cAM^ | 449.54  ±1.58^dBM^ | 519.36  ±2.82^aAN^ | 463.57  ±0.40^cBL^ | 490.44  ±5.08^bAM^ | 449.88  ±3.00^cBM^ | 626.90  ±0.16^aAK^ | 409.86  ±0.04^dBM^ | 505.52  ±9.08^bAL^ | 552.44  ±0.20^bBK^ | 606.71  ±0.90^aBL^ | 538.60  ±2.93^cAK^ | 526.01  ±2.72^dAK^ |
|  | 120 | 436.27  ±0.01^cBM^ | 457.77  ±1.66^aBL^ | 452.79  ±1.55^bAM^ | 438.58  ±1.68^cBM^ | 720.52  ±4.24^aAK^ | 445.78  ±0.50^dBM^ | 528.78  ±0.49^bAK^ | 488.87  ±1.07^cAL^ | 594.87  ±2.48^aAL^ | 361.09  ±0.20^cBN^ | 530.99  ±2.19^bAK^ | 522.08  ±4.60^bAK^ | 601.51  ±0.82^bAL^ | 630.89  ±0.51^aAK^ | 507.88  ±1.35^cBL^ | 437.81  ±0.79^dBM^ |
| Histidine | 0 | 590.24  ±0.47^bAK^ | 606.61  ±2.14^aBL^ | 486.94  ±2.91^cBN^ | 611.96  ±5.47^aAL^ | 582.43  ±2.57^cBL^ | 570.45  ±1.73^dAN^ | 596.16  ±2.70^bBK^ | 650.50  ±5.42^aAK^ | 498.70  ±3.15^cBN^ | 599.87  ±1.76^aAM^ | 525.89  ±1.51^bBM^ | 592.84  ±12.54^aALM^ | 563.91  ±2.35^dBM^ | 643.25  ±0.21^aBK^ | 573.40  ±0.29^cAL^ | 583.22  ±3.23^bBM^ |
|  | 120 | 492.06  ±23.33^dBN^ | 656.95  ±1.64^aAL^ | 613.76  ±0.55^bAM^ | 562.13  ±0.13^cBL^ | 1021.10  ±3.62^aAK^ | 538.36  ±10.91^dAM^ | 673.90  ±0.85^bAK^ | 564.31  ±3.50^cBL^ | 825.55  ±22.85^aAL^ | 439.47  ±6.62^cBN^ | 645.06  ±6.48^bAL^ | 617.10  ±1.38^bAK^ | 715.06  ±5.50^aAM^ | 677.92  ±7.09^bAK^ | 567.83  ±0.95^dBN^ | 617.22  ±2.01^cAK^ |

Mean ± Standard Deviation. ST; Storage Time (days), ERP; encapsulated raspberry powder. Different lowercase letters (a-d) in the same row indicate significant differences between ERP treatments for the same storage day (P<0.05). Different uppercase letters (A-B) in the same column indicate significant differences between storage days for the same treatment (P<0.05). Different uppercase letters (K-N) in the same column indicate significant differences between the initial cemen pH values for the same ERP treatment (P<0.05).
