# Supplementary material for: Properties of encapsulated raspberry powder and its efficacy for improving the color stability and amino acid composition of pastırma cemen pastes with different pH during long term cold-storage
Source: J Food Sci Technol. 2024 Jul 20;62(2):310–24. doi: 10.1007/s13197-024-06029-6 (PMC11757660; doi:10.1007/s13197-024-06029-6)
Supplement: Supplementary file 2 — Supplementary Material 2 [file 13197_2024_6029_MOESM2_ESM.docx]

**Article Type:** Original research article

**Title:** Properties of encapsulated raspberry powder and its efficacy for improving the color stability and amino acid composition of pastırma cemen pastes with different pH during long term cold-storage

**Dear Dr. Aashitosh Ashok Inamdar
Editor
Journal of Food Science and Technology**

Thank you for examining our manuscript (Manuscript Number: **JFST-D-22-01652**)

We would like to thank your and reviewers for valuable contribution to improve our manuscript. All of the required corrections have been made according to the your and referee’s suggestions, it given “Response for Reviewer’s” as detailed. I hope that there is not a problem.

Corrections were made to the R1 form of the article in line with the reviewer’s suggestions, therefore the number of references and total word count increased.

**The total number of words (excluding tables and figure legends) of the manuscript is 7993, the number of references is 50, and the total number of tables and / or figures in the manuscript is 6 and supplemented materials.**

**Highlights**

- This is the first study in which cemen paste (CP) enhanced by using encapsulated raspberry juice powders (ERP) by using spray-dryer
- This is the first study in which ERP main quality and biochemical properties characterized
- CP samples with various initial pH and ERP content were investigated and decreasing pH and increasing ERP significantly improved CP storage stability

**Declaration of Competing Interest**

The author declares that they have no conflict of interest.

**Consent for publication**

All authors have agreed with the contents of this manuscript and submission.

We declare that this manuscript is original, has not been published before and is not currently being considered for publication elsewhere.

I have refereed many articles in your journal. With this, I undertake to review at least three articles submitted to JFST (in my specialization).

Thank you for your understanding and cooperation. I look forward to hearing from you.

Best regards.

**Muhammet Irfan Aksu, PhD, Prof. Dr.**

**Atatürk University, Erzurum/TURKEY**
